# New method for taxonomic descriptions with coded notation, producing dynamic and interchangeable output

**DOI:** 10.1002/ece3.11206

**Published:** 2024-07-04

**Authors:** Douglas Zeppelini, Misael Augusto de Oliveira‐Neto, João Victor Lemos Cavalcante de Oliveira, Aila Soares Ferreira, Roniere Andrade de Brito, Bruna Carolline Honório Lopes, Nathan Paiva Brito, Luis Carlos Stievano, Estevam Cipriano Araujo de Lima

**Affiliations:** ^1^ Laboratório de Sistemática de Collembola e Conservação, Instituto de Biologia de Solo Universidade Estadual da Paraíba João Pessoa PB Brazil; ^2^ Programa de Pós‐Graduação em Ciências Biológicas – Zoologia Universidade Federal da Paraíba João Pessoa PB Brazil

**Keywords:** biodiversity decline, coded species description, Collembola, machine learning, taxonomy

## Abstract

A proposal for taxonomic species description notation is presented to replace the traditional descriptive texts for a coded matrix, avoiding redundant adjectives and subjective descriptions. This is an attempt to enhance the species description rate and to make the descriptions output available to other scientific disciplines, machine learning, interactive and computer‐assisted identification keys, metadata analysis and its applications. The method consists of presenting the description of the overall morphology in a coded matrix, following a character list with detailed observed conditions for each character. The method is dynamic and open to amendments and new data addition as they become available. We test the new method describing five new species of Collembola Symphypleona of the genus *Pararrhopalites* as a generalized model and made the coded output available. We conclude that a coded taxonomic description is an advance to the traditional taxonomic text, with potential to enhance the global descriptions rate. The generated descriptions are dynamic, expandable and can be easily used in other fields of science, allowing non‐experts to access the data for phylogenetic, biogeographic, ecological studies and metadata analysis. Even though an experienced taxonomist will always be necessary to make a detailed taxonomic description, it is a step forward to a general template to semi‐automated taxon recognition and to future development of auxiliary tools for species description using machine learning and templates to speed up the time‐consuming phase of schematic figures preparation, after the expert interpretations are done.

## Graphical abstract

A new proposal for taxonomic description notation, to replace the descriptive text, using a coded list of descriptive characters matched with illustrations. This is an attempt to make the taxonomic information directly available, even to non‐experts, to different kinds of analysis. The proposed method is also a tool for enhancing the rates of taxonomic descriptions for diverse groups, to couple the deficit in the knowledge of the arthropod diversity. The descriptive matrices can be expanded as new information become available and is friendly with machine learning and its applicabilities.

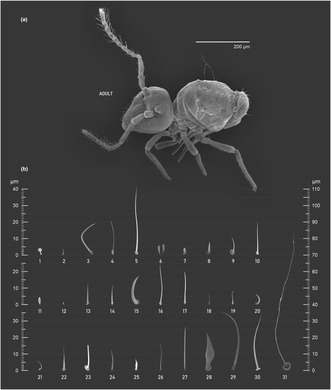

## 1 INTRODUCTION

Taxonomy has been the focus of debate since the XIX century, and even recently the recognition of the taxonomic research is subject of discussion (Packer et al., 2018; Zeppelini et al., 2021). The global biodiversity crisis exposes the urgency for investment in taxonomy to reveal the largely unknown species diversity. Using Collembola as a parameter, where about 20% of its estimated diversity is known (Hopkin, 1997), between 100 and 120 new species are described each year, and it would take to taxonomists more than 400 years to uncover and describe all the unknown species diversity (Potapov et al., 2020). To be able to understand the diversification processes in Collembola, we need to speed up the rates of species description. This is a matter of concern in every area of entomology, and in some extent, the whole zoology.

Collembola Lubbock, 1870 are minute wingless arthropods, basal hexapods found in every terrestrial habitat on the planet, including soil, leaf litter, canopy trees and caves (Bellinger et al., 1996; Hopkin, 1997). There are about 9000 described species, and its diversity is extensively underestimated and poorly known (Bellinger et al., 1996, Hopkin, 1997). They play important role in the soil food web and the global metabolism (Bardgett & van der Putten, 2014; Filser et al., 2016; Potapov et al., 2023; Rusek, 1998).

Similar to many other taxonomic groups of meso‐ and micro‐fauna, Collembola taxonomy is largely based on morphological analysis, observing, and describing discrete variations in diagnostic characters. The most abundant morphological source of information for species definition in Collembola is the number, distribution, and shape of cuticular chaetae, this is called chaetotaxy. The current morphological approaches for inference of homology, chaetotaxic systems for chaetal identification, are often room for great subjectivity depending on what is seen and what is visible under an optic microscope, and often different chaetotaxy systems are hardly comparable (Betsch, 1997; Betsch & Waller, 1994; Bretfeld, 1990, 1999; Potapov et al., 2020). The challenges and perspectives for Collembola taxonomy is discussed in detail, and the need for an integrative taxonomy and international efforts to direct financial support and expertise recognition to face the global biodiversity crisis, was also the focus of debate (Potapov et al., 2020; Zeppelini et al., 2021).

The impact of recent technologies of high‐resolution imaging, molecular sequencing and machine learning will be a great deal towards taxonomic techniques that can improve new and known taxa recognition (Potapov et al., 2020). Integrative taxonomy, combining morphological and molecular data to define species limits is likely to be a trend for most taxonomic groups, not only Collembola.

There is, however, a particular aspect in Collembola (and nearly every taxon of the meso‐ and micro‐fauna) that affects the viability of including molecular sequences in new species descriptions, in many, if not most cases. It is rather a logistic problem, but many times there is not an alternative. The problem is that almost all new species are discovered under light microscope, which means that the specimen was mounted in a slide, after being cleared under several different techniques of chemical washes, which destroy the tissues and, consequently, genetic material.

It is only after the taxonomic identification, that a species is recognized as new for science or undescribed. More often than not, the material analyzed is a limited set of specimens, and there is no available material for molecular analysis after the taxonomic identification and morphologic study, if the extraction of DNA/RNA was not performed before mounting the specimens in slides for microscopy. Accepting that molecular analysis facilities are available, many times the biological specimens needed for molecular sequencing may be available only in a future, after the species is described. Even when scanning electron microscopy (SEM) is possible, depending on the structure, it is hard to get images of all diagnostic features and light microscopy may be needed as well. Nevertheless, high‐resolution imaging and molecular data are powerful tools, and may be indispensable for accurate taxonomic research and species delimitation. Therefore, the morphologic descriptions must be dynamic, open to easy amendment and additional data insertion. Furthermore, it must be presented in an interchangeable language, to allow the information to flow across different disciplines.

Among all methods applied to the external morphology study of Collembola, chaetotaxy is certainly the most complex and extensively detailed (Betsch & Waller, 1994; Cassagnau, 1974; Deharveng, 1983; Fjellberg, 1999; Jordana & Baquero, 2005; Nayrolles, 1988, 1990a, 1990b; Potapov, 2001; Szeptycki, 1979, 1972; Yosii, 1960). There are many chaetae and groups of chaetae that vary in position and shape in such a way that they allow a great deal of homology inferences. However, the most advanced approaches are also very complex, which make interpretation difficult and increase ambiguity. These aspects circumscribe the deep taxonomic research to restricted groups of experts, posing difficulties to comparative studies even among different orders of Collembola. In addition, the traditional descriptive texts with morphological and chaetotaxic information are difficult to integrate with machine learning and computational novelties, which could give a lot of agility to phylogenetic analysis, metadata comparison, biogeography, and their various applications (Potapov et al., 2020).

Despite all advances in technological instruments and methods, taxonomic descriptions are still written basically in the format as it was about two centuries ago, with a hermetic language in nearly incomprehensible texts for non‐experts. This is often a greater barrier to communication among different areas of science, than the access of high‐tech equipment and analytical facilities.

The proposal of a coded and illustrated description of new species that can be easily imported, transformed, amended, corrected, or expanded is presented as an alternative to the traditional descriptive taxonomic method.

The strength of the coded description is that new characters, whether morphological, molecular, and ecological, can be easily added to the list and can improve the descriptive matrix as new information is produced. These matrices can be uploaded to public libraries and kept up to date with all available information about the species, and linked to data bases as GBIF, ZooBank and electronic taxonomic catalogs available in different parts of the world, e.g., fauna.jbrj.gov.br/fauna/listaBrasil (Zeppelini et al., 2023) and www.collembola.org (Bellinger et al., 1996).

Finally, the new proposition for taxonomic notation will not dismiss the need of a experienced taxonomist, as the *pre* and *post* descriptional elements (e.g., type material, habitat, distribution, remarks), and all the analytical study (morphological, molecular) will always depend on the expertise of the researcher, nevertheless the identification phase can be automatized and the schematic figures preparation, a very time‐consuming phase of the whole description work, can be speed up with templates for each taxonomic group in a pop up fashion, as the data matrix is fulfilled. This may allow the taxonomists to enhance their productivity, increasing the species descriptions rate.

## 2 MATERIALS AND METHODS

### 2.1 Coded taxonomic description

The order Symphypleona Börner, 1901 shows some ambiguity in current morphological methods, particularly when describing the head and body chaetotaxy (Betsch, 1997; Betsch & Waller, 1994; Bretfeld, 1990, 1999; Christiansen & Bellinger, 1998). The order is composed by springtails with globular body shape, as a result of modification and fusion of thoracic and abdominal segments I–IV, this condition hinders the direct assignment of segments identity.

An approach that can reduce the ambiguity of the taxonomic descriptions is the description of body parts into coded morphological units, straightforwardly representing the actual body segments and appendicular whorls (Hopkin, 1997; Jura et al., 1987; Nayrolles, 1988, 1990a, 1990b, 1991; Tomizuka & Machida, 2015), in such a way that any species can be compared from the coded data base. This is in replacement to the traditional descriptive text, many times with ambiguous terminology, and often applying different and not directly comparable chaetotaxic systems. The coded notation method would lead to a more comprehensive analysis of the chaetotaxy, as well as a direct availability of the data for comparative studies. Furthermore, a coded description can easily be amended, molecular data can be added in the character list and matrix, and new complementary morphological features can be inserted as new information is available.

The qualitative description of the shape and size of the different chaeta is also subject to a great deal of ambiguity and poor definition, the adjectives are not standard and the very definition of what is a macro‐, meso‐ or microchaeta is not always clear. Therefore, a bank of shapes with high quality images is imperative to discard all the subjective descriptions. There are several chaetae banks published for different groups of Collembola, including some with precise line drawings (Betsch, 1980; Christiansen, 1966; Deharveng, 1983; Nayrolles, 1991), and some with SEM photography (Cipola et al., 2020; de Lima et al., 2022; Lukić et al., 2010; Zeppelini et al., 2022; Zhang & Deharveng, 2015), it is a matter of time to have a fully reliable chaetal shapes collection, so a specific chaeta can be addressed directly by its reference in the bank, in the coded description.

A standard, fully coded method for species description may be an improvement to the traditional descriptive text, it may allow to use machine learning and high‐quality imaging to enhance the efficiency of species descriptions and diversity recognition, offering a powerful tool to understanding of global processes of diversification and distribution, and face the biodiversity decline.

### 2.2 Chaetal fields and morphological units

**FIGURE 1:**
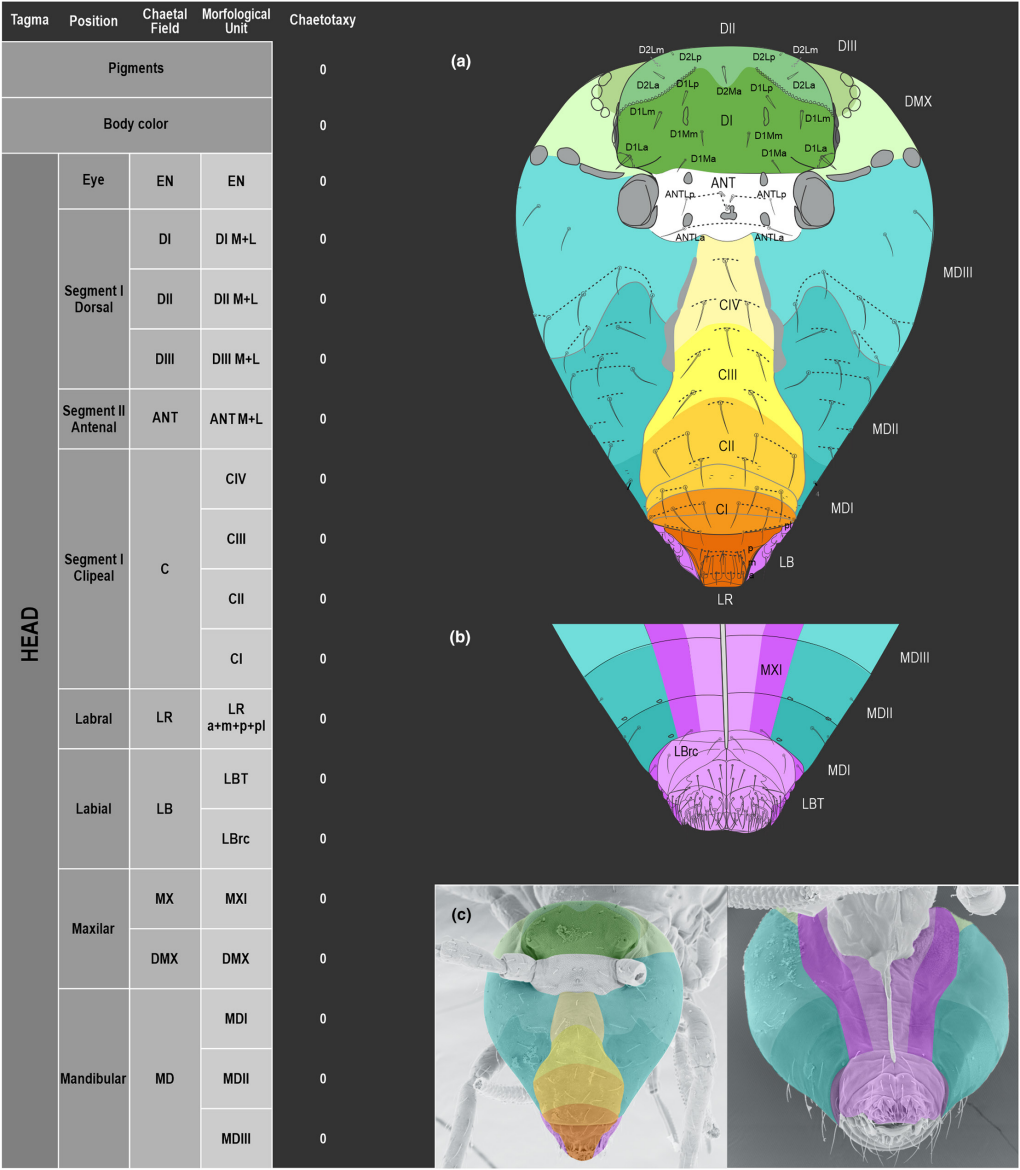
*Pararrhopalites fallaciosus* **sp. n.** cephalic chaetotaxy and descriptive table. (a) Dorsal cephalic schematic chaetotaxy; (b) ventral cephalic schematic chaetotaxy; (c) dorsal and ventral cephalic SEM. Abbreviations in the descriptive table as in Table 1.

**FIGURE 2:**
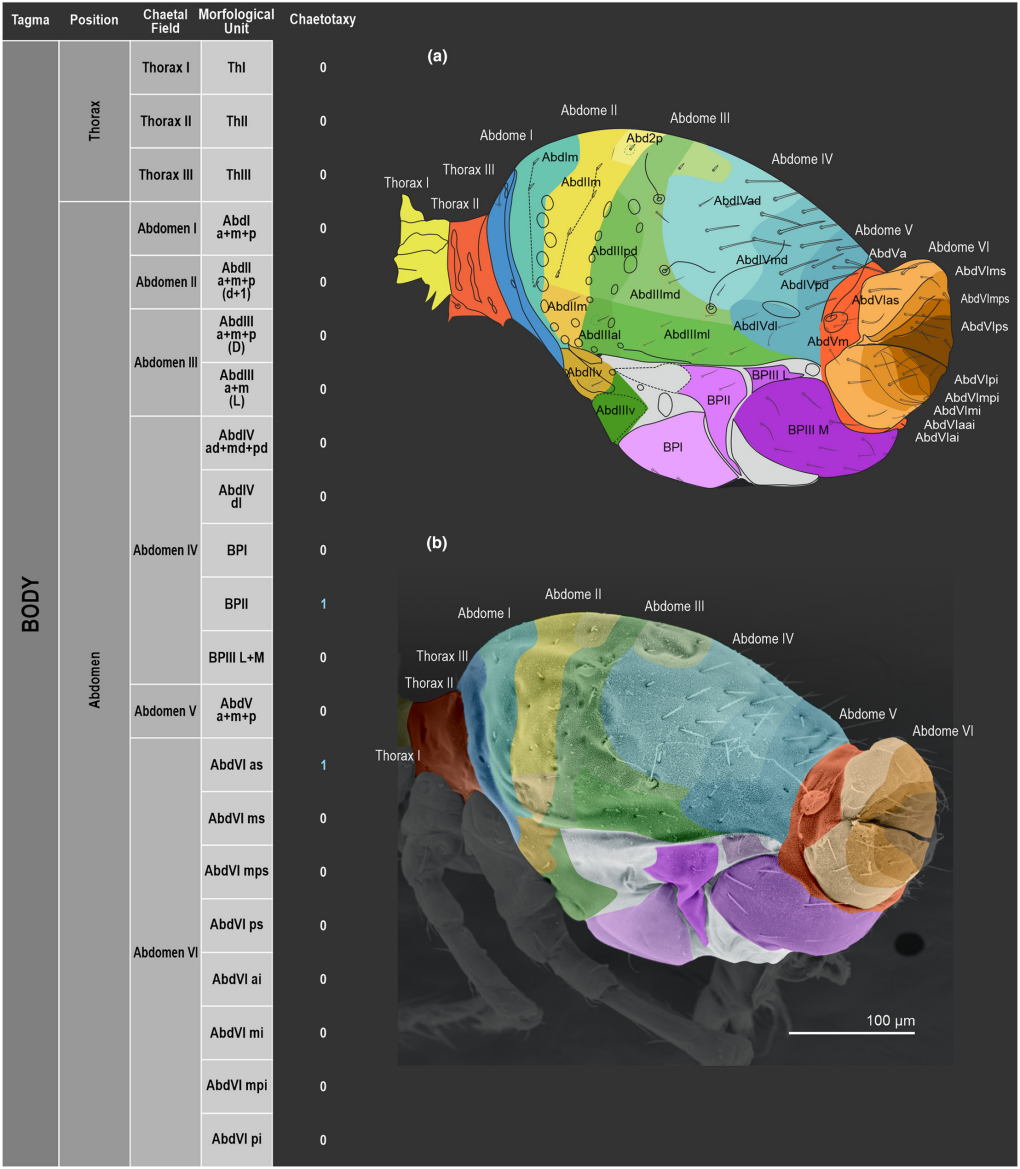
*Pararrhopalites fallaciosus* **sp. n.** body chaetotaxy and descriptive table. (a) Whole body schematic chaetotaxy; (b) whole body SEM.

**FIGURE 3:**
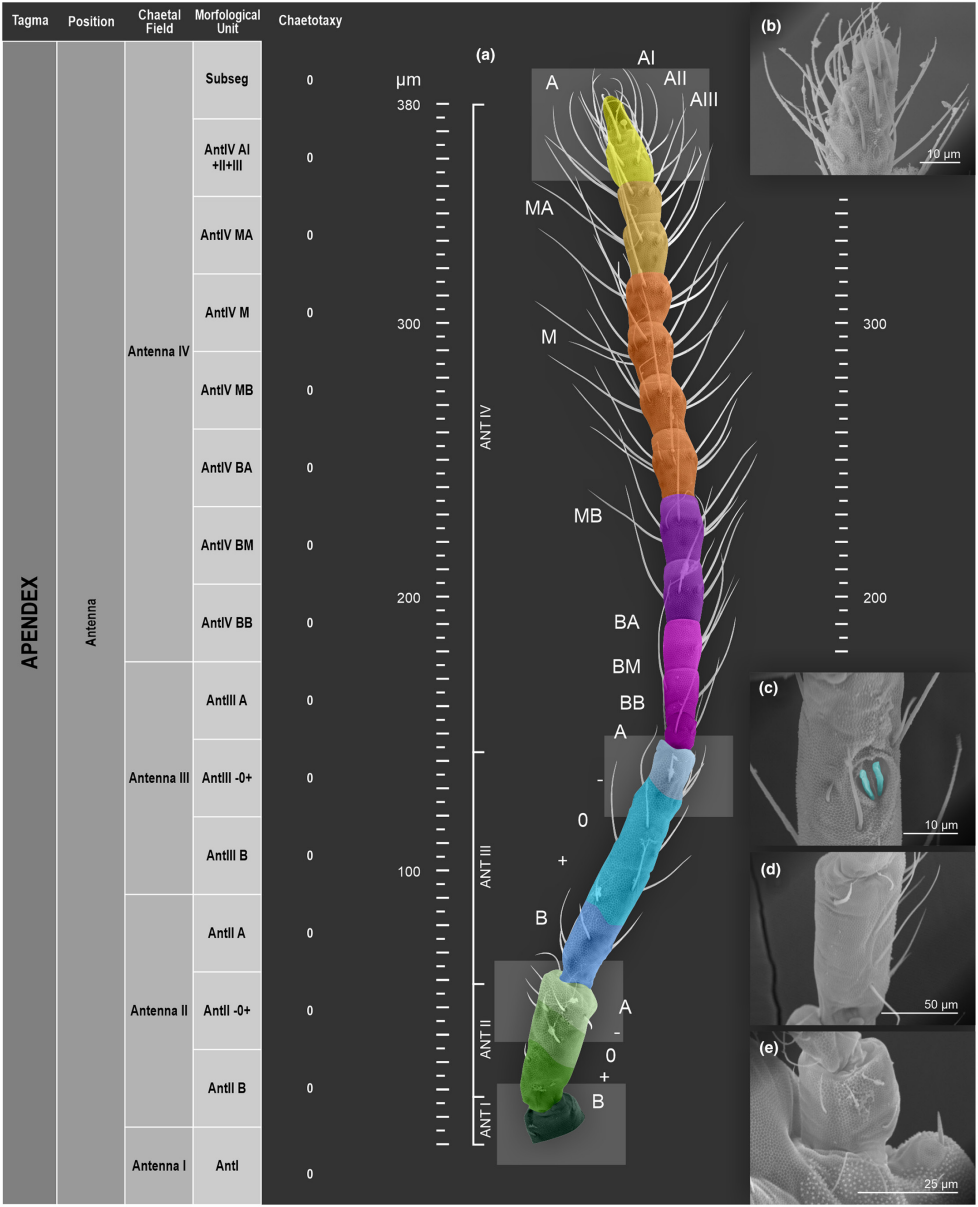
*Pararrhopalites fallaciosus* **sp. n.** antennal chaetotaxy SEM and descriptive table. (a) Whole antennal chaetotaxy; (b) Ant. IV apical subsegment; (c) apical organ on Ant. III; (d) Ant. II segment mid‐posterior view; (e) Ant. I mid‐dorsal view.

**FIGURE 4:**
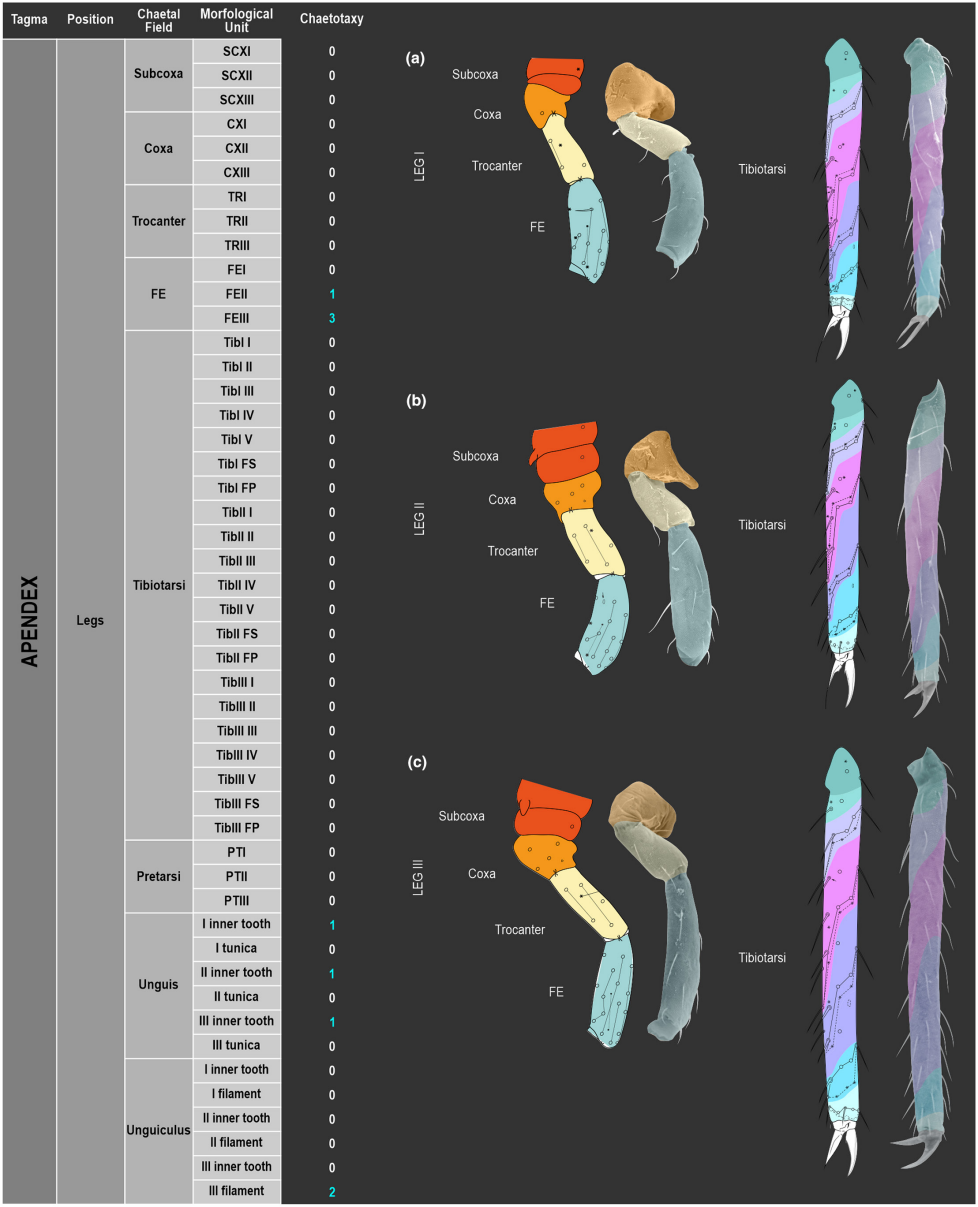
*Pararrhopalites fallaciosus* **sp. n.** legs chaetotaxy and descriptive table. (a) Leg I; (b) leg II; (c) leg III. For each represented leg, the left side is a schematic chaetotaxy, the right side is SEM.

**FIGURE 5:**
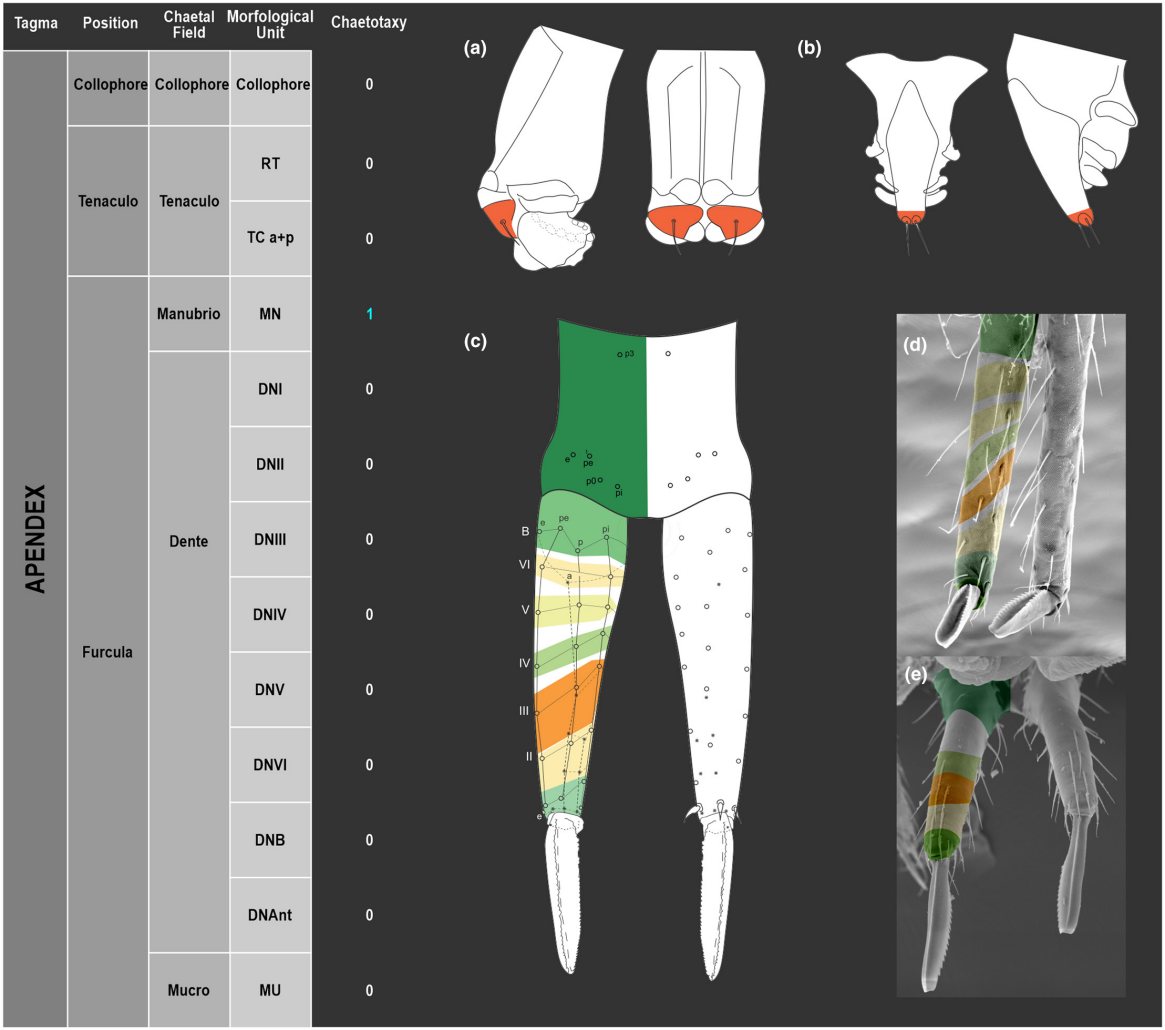
*Pararrhopalites fallaciosus* **sp. n.** Abdominal appendages chaetotaxy. (a) Ventral tube lateral view and anterior view; (b) tenaculum anterior view and lateral view; (c) furcula schematic chaetotaxy (solid circles – anterior view; hollow circles – posterior view); (d) dens and mucro, posterior view, SEM; (e) dens and mucro, anterior view, SEM.

**FIGURE 6:**
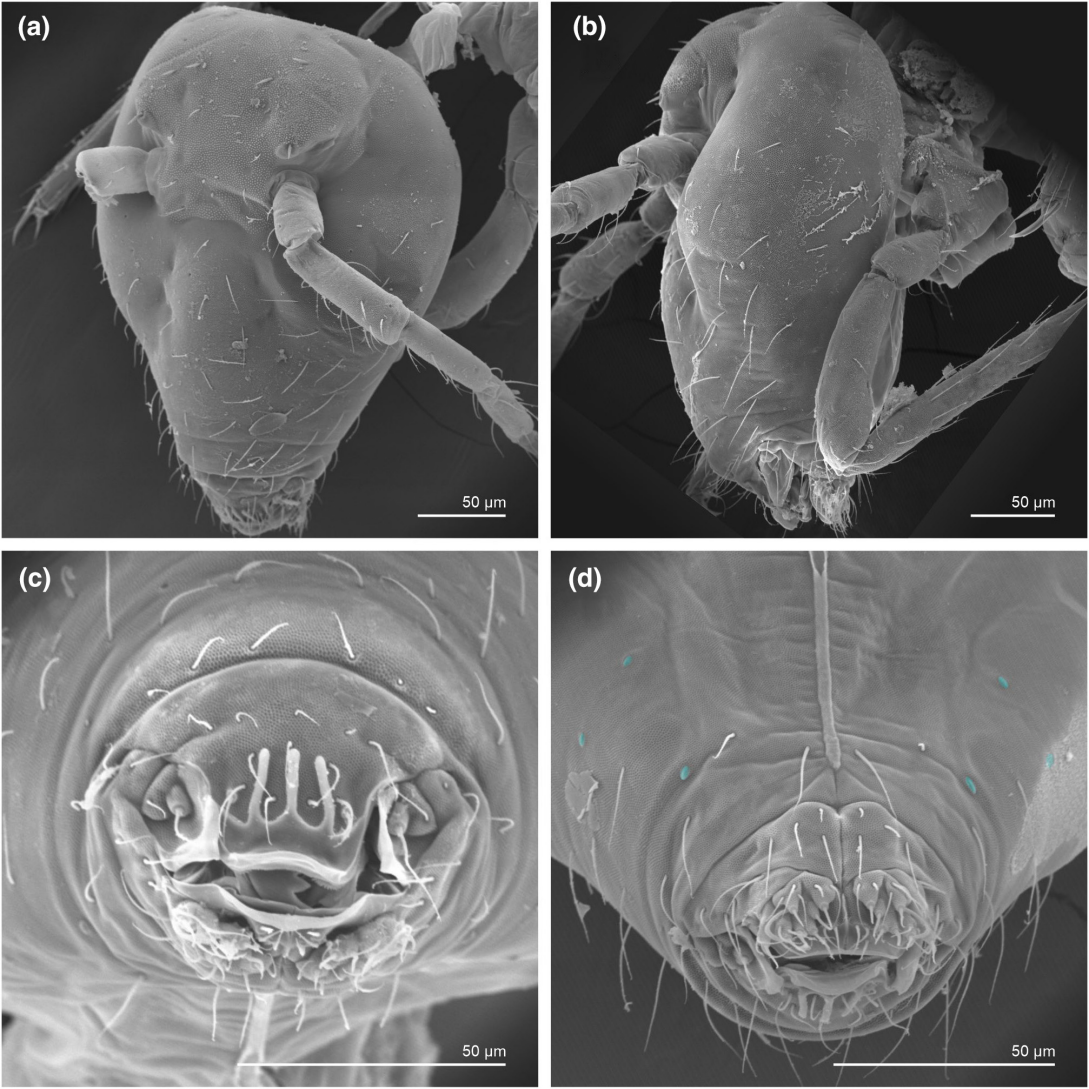
*Pararrhopalites fallaciosus* **sp. n.** SEM of the head showing the chaetotaxy and cuticular landmarks. (a) Dorsal view; (b) lateral view; (c) labrum; (d) labium and adjacent chaetae (oval organs in blue).

**FIGURE 7:**
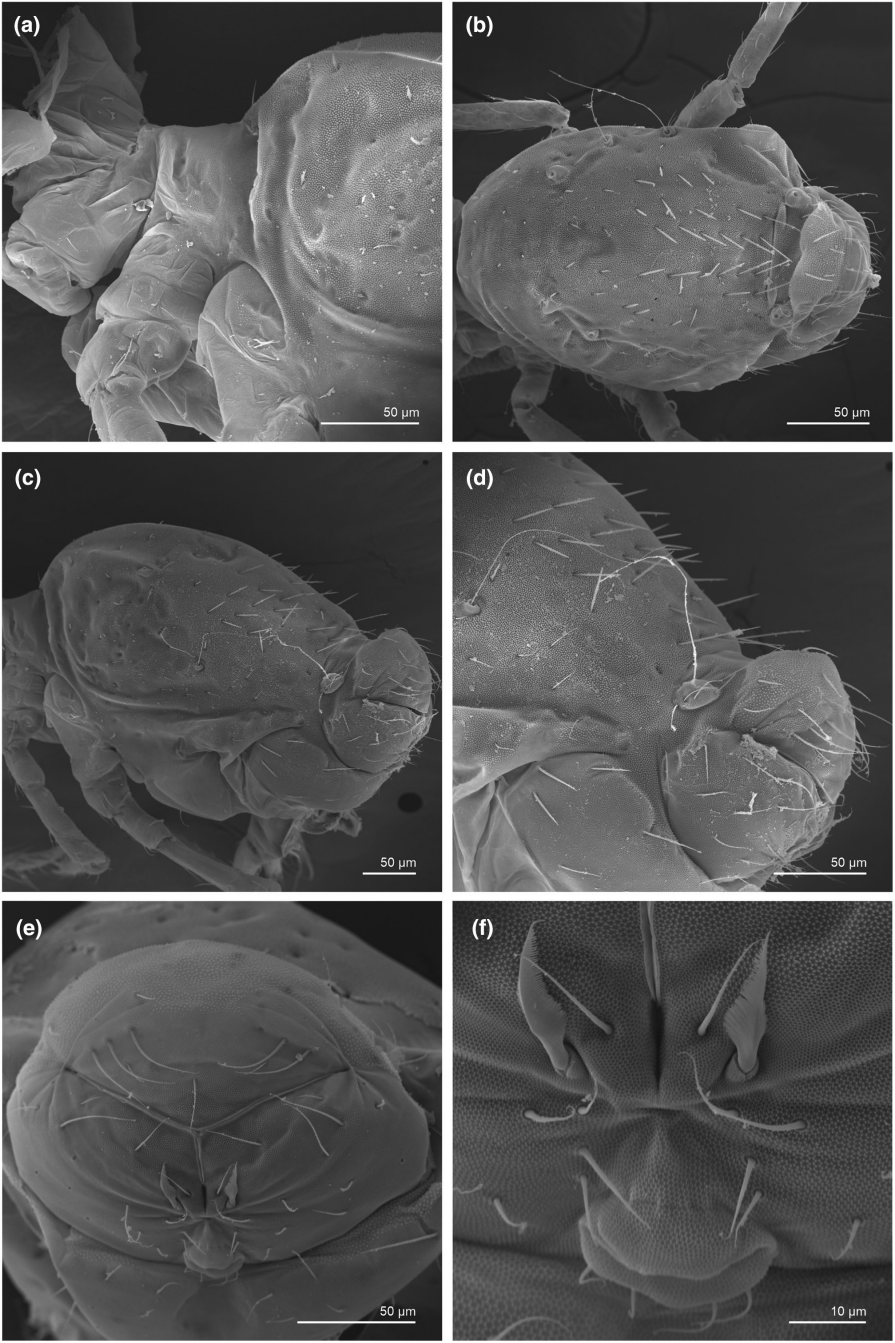
*Pararrhopalites fallaciosus* **sp. n.** SEM of the body showing general chaetotaxy and cuticular landmarks. (a) Thorax and abd. I–II lateral view; (b) whole abdomen dorsal view; (c) whole abdomen lateral view, (d) abd. IV–VI lateral view; (e) female anal valve posterior view; (f) female subanal appendages and genital opening.

We attempt to access the chaetotaxy of the head and great abdomen of Collembola, by identifying body segments arranged in each tagma (Figures 1 and 2), or whorl in each appendage (Figures 3, 4, 5) (Hopkin, 1997; Jura et al., 1987; Nayrolles, 1988, 1990a, 1990b, 1991; Tomizuka & Machida, 2015). Each segment has its own set of chaetae, and more than one chaetal field may be observed in a single body segment. A chaetal field is a group of associated chaetae that are consistently observed in a given body segment, often associated with some landmark on the cuticle (Figures 6 and 7).

The morphological units are the actual observed character in a given species. Once recognized all the chaetal fields, the characters are listed, and their inherent character states are described. Each morphological unit in the character list is given a code (0, 1, 2) to each observed condition. It is important to note that it is not a phylogenetic matrix, once the codes in the resulting matrix are not supposed to include hypothesis of ordering or polarity, and both apomorphies and plesiomorphies may be listed as character, instead, it is a descriptive coded character state matrix.

### 2.3 Head, body, and appendages chaetotaxy

Chaetotaxic systems attempt to labeling each chaeta along the body, where the label indicates a specific chaeta and its position in the body of the animal, then a qualitative description is made (e.g., spinelike chaeta, macrochaeta, club shaped sensillum, palmate, serrate, lanceolate), bringing subjectivity in the interpretation of a specific chaeta labeling, and by the many different adjectives that can apply for a given shape, depending on the author.

**FIGURE 8:**
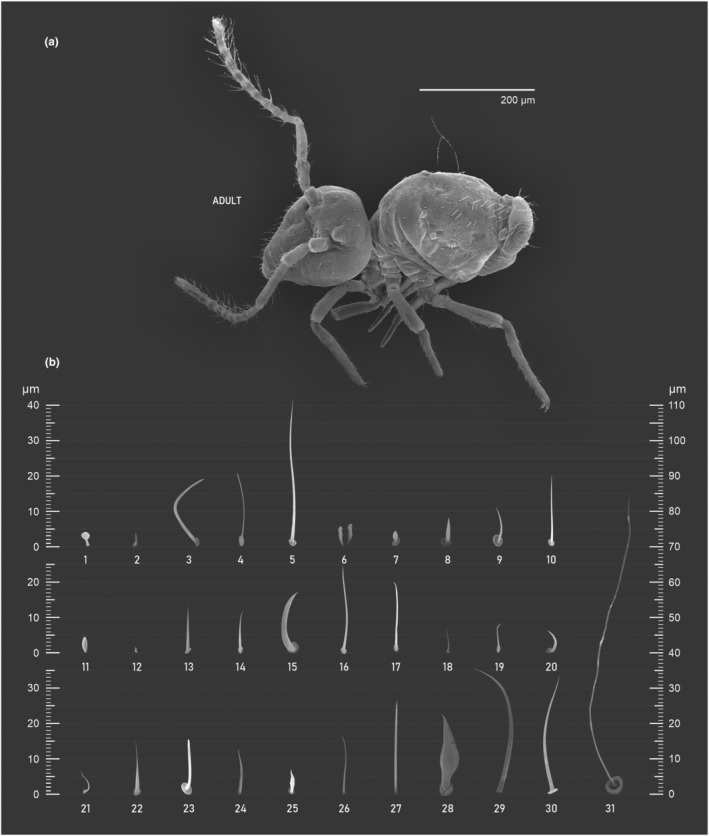
Chaetae bank, it is a collection of SEM photographs of different chaetae found along the body, the chaetae are representatives of their shapes and sizes, but not of their position in the body, therefore a given shape and size represented by a photo in the bank may occur in different parts of the body. (a) Habitus *Pararrhopalites fallaciosus* **sp. n.**; (b) chaetae bank. 1 – Bulb sensillum (usually found in the apex of ant IV); 2 – Microsensillum (e.g., Aai on the apical organ of Ant. III); 3 – Macrochaeta, curved with blunt tip (often associated to bulb sensillum on the apex of Ant. IV); 4 – Bristlelike mesochaeta; 5 – Smooth macrochaetae; 6 – Microsensillum with blunt apex (occurs in pairs on the Ant. III apical organ); 7 – Semi globular microsensillum; 8 – Spiniform mesochaeta; 9 – Smooth curved mesochaeta; 10 – Smooth stout macrochaeta; 11 – Oval organ; 12 – Pre tarsal spine (other groups present a microchaeta instead); 13 – Slender spinelike mesochaeta; 14 – Smooth stout mesochaeta; 15 – Strongly spinelike macrochaeta (e.g., metatrochanteral spine); 16 – Slender smooth macrochaeta; 17 – Smooth strong macrochaeta (similar to 10); 18 – Slender smooth microchaeta; 19 – Smooth stout microchaeta; 20 – Curved spinelike microchaeta; 21 – Bristlelike microchaeta; 22 – Spinelike mesochaeta; 23 – J‐shaped blunt mesosensillum; 24 – Smooth mesochaeta; 25 – Heavily spiniform mesochaeta (similar to 8); 26 – Slender smooth mesochaeta; 27 – Smooth broad macrochaeta (similar to 10 and 17, but longer); 28 – Featherlike macrochaeta (subanal appendage); 29 – Broad lanceolate macrochaeta (subanal appendages, sometimes ciliated); 30 – Broad smooth macrochaeta; 31 – Trichobothria.

Here we map regions that can be compared in different taxonomic groups, the chaetal fields, within each head and body segment (Figures 1 and 2), and to appendages (Figures 3, 4, 5), and refer to a shape for the chaeta in an image data set, the chaetae bank, with images of each kind of chaeta found in the taxon (Figure 8).

After the analysis of the chaetotaxy applying the traditional systems, the labeling of the chaetae is replaced by a code describing the total number of chaetae in the chaetal field, and the qualitative description of the different kind of chaetae found in each chaetal field, is replaced by the respective number in the chaetae bank that represents the actual observed shape and size, to compose the morphological unit definition (see Table 1).

**TABLE 1:** Morphologic description of *Pararrhopalites fallaciosus* **sp. n**.

Tagma	Segment	Chaetal field	Morphologic unit	Morphologic unit state
Head			Pigments	−
			Body color	White
	Eye	Eyes number	EN	1+1
	Dorsal	D	DI M + L	5+5 4(9)6(8)
			DII M + L	3+1+3 7(8)
			DIII M + L	0+0
	Antennal	ANT	ANT M + L	3+1+3 4(9)3(7)
	Clypeal	C	CI	3+3 6(10)
			CII	5+1+5 11(10)
			CIII	2+1+2 5(10)
			CIV	1+1+1 3(10)
	Labral	LR	LR a + m + p + pl	4+5+5+6 20(10)
	Labial	LB	LBT	4+4 8(10)
			LBrc	2+2 4(10)
	Mandibular	MD	MDI (d + v)	2+2+1+1ov 4(10)2(11)
			MDII (d + v)	8+8+2+2ov 16(10)4(11)
			MDIII (d + v)	10+10 20(10)
	Maxilar	MX	DMX	0+0
			MXI	2+2 4(10)
Body	Thorax	Thorax I	ThI	0+0
		Thorax II	ThII	1+1 2(24)
		Thorax III	ThIII	3+3 2(24)2(26)2(23)
	Abdomen	Abdomen I	AbdI a + m + p	3+3 6(25)
		Abdomen II	AbdII a + m + p (d + l)	8+8 8(25)8(26)
		Abdomen III	AbdIII a + m + p (d)	10+10 8(25)6(26)6(31)
			AbdIII a + m (l)	5+5 10(26)
		Abdomen IV	AbdIV ad + md + pd	17+17 4(25)30(27)
			AbdIV (l)	3+3 6(26)
			BPI	2+2 4(26)
			BPII	1+1 2(26)
			BPIII l + m	13+13 10(26)16(18)
		Abdomen V	AbdV a + m + p	7+7 12(26)2(31)
		Abdomen VI	AbdVI as	4+4 8(26)
			AbdVI ms	2+2 4(26)
			AbdVI mps	3+1+3 7(30)
			AbdVI ps	1+1+1 3(26)
			AbdVI aai + ai	8+8 16(26)
			AbdVI mi	3+3 6(26)
			AbdVI mpi	4+4 6(26)2(28)
			AbdVI pi	3+3 6(26)
Appendages	Antenna	Antenna I	AntI	6 5(9)1(21)
		Antenna II	AntIIA	7 6(9)1(21)
			AntII −0+	3 3(9)
			AntIIB	4 4(9)
	
		Antenna III	AntIIIA	8 2(6)1(2)1(21)1(20)3(9)
			AntIII −0+	8 4(9)4(5)
			AntIIIB	4 4(9)
		Antenna IV	Subsegments	10
		Number of chaeta/whorl	AntIVAI + II + III	34 1(3)1(1)2(4)6(10)24(5)
			AntIVMA	8 1(4)6(5)
			AntIVM	9 1(4)8(5)
			AntIVMB	8 1(4)7(5)
			AntIVBA	3 3(5)
			AntIVBM	3 3(5)
	AntIVBB		4 4(5)
	Legs	Subcoxa	SCXI	1 1(26)
			SCXII	1 1(26)
			SCXIII	2 2(26)
		Coxa	CXI	1 1(26)
			CXII	3 3(26)
			CXIII	5 5(26)
		Trocanter	TRI	5 5(26)
			TRII	6 6(26)
			TRIII	6 1(15)5(26)
		Femur	FEI	15 15(26)
			FEII	14 14(26)
			FEIII	12 12(26)
		Tibiotarsus	TibI I	9 1(13)8(14)
			TibI II	8 1(13)7(16)
			TibI III	8 1(13)7(16)
			TibI IV	14 1(13)13(16)?
			TibI V	8 1(13)7(16)
			TibI.FS	2 2(16)
			TibI.FP	3 3(16)
			TibII I	9 1(13)8(14)
			TibII II	8 1(13)7(16)
			TibII III	8 1(13)7(16)
			TibII IV	14 1(13)13(16)
			TibII V	8 1(13)7(16)
			TibII FS	3 3(16)
			TibII FP	3 3(16)
			TibIII I	9 1(13)8(14)
			TibIII II	8 1(13)7(16)
			TibIII III	8 1(13)7(16)
			TibIII IV	14 2(13)12(16)
			TibIII V	8 1(13)7(16)
			TibIII FS	3 3(16)
TibIII FP			3 3(16)


		Pretarsus	PTI	1+1 2(12)
			PTII	1+1 2(12)
			PTIII	1+1 2(12)
		Unguis	Unguis I inner tooth	−
			Unguis I tunica	−
			Unguis II inner tooth	−
			Unguis II tunica	−
			Unguis III inner tooth	−
			Unguis III tunica	−
		Unguiculus	Unguiculus I filament	>
			Unguiculus I tooth	+
			Unguiculus II filament	>
			Unguiculus II tooth	+
			Unguiculus III filament	<
			Unguiculus III tooth	+
	Collophore	Collophore	1+1 2(19)
	Tenaculo	Tenaculo	RT	3+3 teeth
			TC a + p	2 2(18)
	Furcula	Manubrio	MN	5+5 10(16)
		Dente	DNI	5 1(16)4(22)
			DNII	3 3(16)
			DNIII	3 3(16)
			DNIV	3 3(16)
			DNV	3 3(16)
			DNVI	2 2(16)
			DNB	4 4(16)
DNAnt			4,2,2,1,1
Mucro		Mucro	Both lamellae serrated

*Note*: The table brings the state of each morphologic unit observed in the species. The code for each state is presented in the character list (Appendix). (*N*) Numbers in parentheses represent the shape of the chaeta in the chaetae bank (Figure 8).

Abbreviations: a, anterior; Abd, abdomen; Ant, antennal segment; C, clypeus; CX, coxa; D, dorsal head; d, dorsal; DN, dens; EN, eyes number; FE, femur; L, lateral dorsal head; l, lateral; LBrc, labium remaining chaetae; LBT, labial triangle; LR, labrum; M, medial dorsal head; m, medial; MD, mandibular segment; md, mid‐dorsal; MN, manubrium; MX, maxilar segment; ov, oval organ; p, posterior; pd, postero‐dorsal; pl, pre labral; PT, pretarsus; SCX, subcoxa; TC, tenacular corpus; Th, thorax; Tib, tibiotarsus; RT, ramus tenacular; TR, trochanter; v, ventral; “−”, absent; “+”, present, “>”, bigger than; “<”, shorter than.

### 2.4 Coded descriptive dataset

The information of the whole collection of data of each species, result in a dataset as synthesized in Table 1, this is the final morphologic description outcome and represents the complete and up to date set of information for each studied species. However, it is dynamic and open to additional information, when available.

The coded dataset is hierarchically ordered in four columns namely, Tagma, Segment, Chaetal field and Morphological unit (Table 1), a fifth column is inserted with the coded information of the species (Figure 1). The lines of the resulting dataset bring the different features of the chaetotaxy and general morphology (e.g., eyes, foot complex). The cells in the column of morphological unit are the actual features to be observed in the specimen, where each one is a recognizable morphological unit of the animal whole morphology, that is described and coded in the character list (Appendix).

Using an example of a defined character and its code according to Appendix, we have: Antennal/ANT/ANT M + L/3+1+3 4(9)3(7), were Antennal indicates the segment, ANT is the chaetal field (see Figure 1a), ANT M + L is the morphologic unit, 3+1+3 are the observed chaetae in the respective chaetal field (3 paired and 1 axial according to bilateral symetry), and 4(9)3(10) means that there are four type “9” chaetae, and three type “7” chaetae (total 7 chaetae or 3+1+3), the types are defined by comparison among the images in Figure 8. To the combination of chaetae number and type is given a numeric code (this example is coded 0 in the character list Appendix).

### 2.5 Testing the coded description

To test the proposed system, we describe five new species of the genus *Pararrhopalites* Bonet & Tellez, 1947 (zoobank.org:pub:9ED865EA‐F95A‐4CBE‐947C‐3A5C6CD81907), from the order Symphypleona, using *Pararrhopalites fallaciosus* **sp. n.**, as the explanatory example in the SEM overall morphologic analysis. First, we describe *P. fallaciosus* **sp. n**., where all the chaetal fields are delimited, and the species is morphologically defined. The character list is derived from this revision and presented in Appendix. The final descriptions for the remaining new species will bring the code only (Morphologic unit code).

Here we propose the coded description for *Pararrhopalites*, a genus of Symphypleona, however, once fully established, the system must be applicable to the orders Poduromorpha and Entomobryomorpha as well. Ideally a similar approach could be used to any other zoological taxa.

To proceed the segmental delimitation and definition, we addressed the results from developmental biology (Hopkin, 1997; Jura et al., 1987; Tomizuka & Machida, 2015), and the chaetal fields were defined using SEM (Tescan Vegas III) to map the clusters of chaetae associated to cuticular landmarks. Cephalic chaetotaxy, great abdomen chaetotaxy, and small abdomen (abdominal segment VI) chaetotaxy followed the system proposed by Betsch and Waller (1994) and Betsch (1997). For the appendages, we followed Nayrolles (1988, 1990a, 1990b, 1991) for antennae, legs and furcula; and tenaculum after Richards (1968).

The chaetotaxic systems were used to verify the congruence of the morphological landmarks and associated groups of chaetae which display constant expression (i.e., chaetal fields). All chaetotaxic information, the actual observed character condition, was described in the character list, and coded accordingly (Appendix).

The descriptions are presented as a list of coded characters in the descriptive plate of each newly described species. The exception is made to *P. fallaciosus* **sp. n.**, where the morphologic units are described in the matrix corresponding to the chaetotaxy system cited above, as an example of what the observed features are (before coding). The detailed chaetotaxy analysis for all the five species described here is available in the Data S1.

## 3 RESULTS

### 3.1 Coded description *Pararrhopalites fallaciosus* sp. n.

**Collembola Lubbock,** **1870**

**Symphypleona Börner,** **1901**

**Sminthuroidea Bretfeld,** **1994**

**Sminthuridae Lubbock,** **1862**

**Sminthurinae Lubbock,** **1862**

**Pararrhopalites Bonet & Tellez,** **1947** **(=Neosphyrotheca Salmon,** **1964** **)**

### 3.2 *Pararrhopalites fallaciosus* sp. n.

zoobank.org:act:C9126B2C‐EB0F‐4AC3‐BD10‐066A54998541

(Figures 1, 2, 3, 4, 5, 6, 7, 8)

**Type material.** Holotype female in slide (#20441/CRFS/UEPB): Brazil, Minas Gerais state, Caeté municipality, F‐29 SSH, 19°49′22.18″ S 43°41′51.61″ W, 26.IV.2022, Ativo ambiental et al. coll. Paratypes in slides (#20439, #20440/CRFS/UEPB): 2 females, same data as holotype.

Additional materials https://doi.org/10.15468/pbxmgz

**Etymology.** *Pararrhopalites fallaciosus* **sp. n.** is a reference to the similarities with a *P. queirozi* Brito et al., 2019, that can lead to an elusive identification.

#### 3.2.1 Habitat and distribution

The species was collected in drilling holes, occurring in the Subterranean Shallow Habitat (SSH), its known distribution is restricted to the type locality, despite the sampling efforts in nearby areas and in the whole region, an important mining area which is being consistently sampled in the last decade.

Good's Biogeographic zone 27 (Culik & Zeppelini Filho, 2003; Good, 1974). The climate according to Köppen's system is As (de Sá Júnior et al., 2012; Köppen, 1936; Shear, 1966), presenting dry winters and wet summers, average temperatures of 18°C during winter and 22°C in summer (valid for all the five species described here).

#### 3.2.2 Remarks

The new species resembles *P. queirozi* in the shape of the subanal appendage and cephalic chaetotaxy but can be clearly distinguished by the number of subsegments of Ant. IV (eight in *P. queirozi*, 10 in the new species), the presence of inner tooth in all ungues, and apical filament exceeding the tip of the unguis in all three empodial complexes in *P. queirozi*. The new species is similar to *Pararrhopalites hermesi* **sp. n.** in the shape of subanal appendages, the lack of inner tooth of all ungues. They differ by the number of eyes (1+1 in *P. fallaciosus* **sp. n.** and 0+0 in *P. hermesi* **sp. n.**), number of subsegments in Ant. IV (10 and nine respectively), the lack of corner tooth in all unguiculi and mucro with inner lamella smooth in *P. hermesi* **sp. n.** The reduced number of chaetae on the dorsal posterior part of the great abdomen (17+17) also differentiates *P. fallaciosus* **sp. n.** from other species of the queirozi‐group (all the species which share the same female subanal appendages).

### 3.3 *Pararrhopalites hermesi* sp. n

**FIGURE 9:**
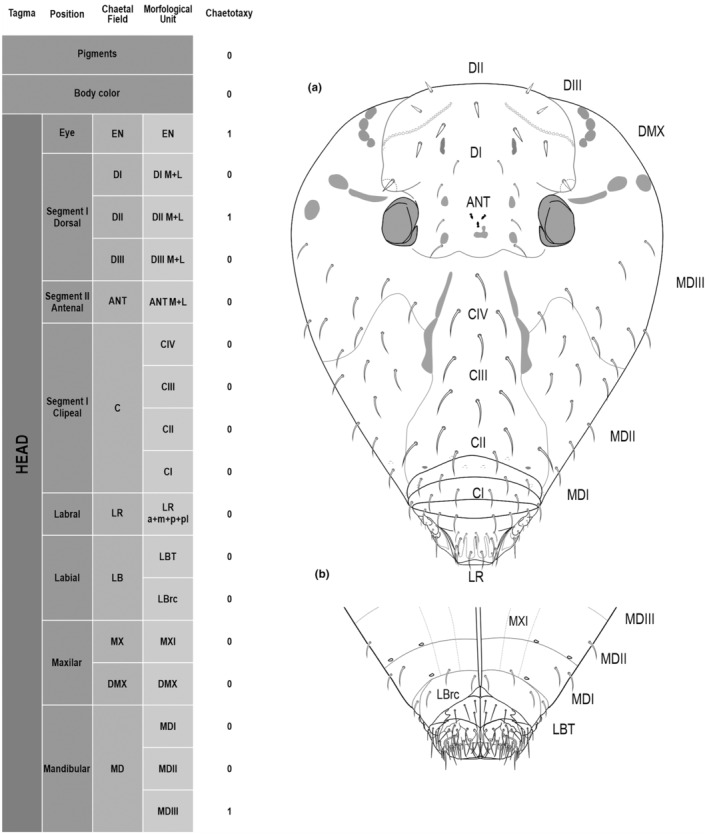
*Pararrhopalites hermesi* **sp. n.** cephalic chaetotaxy and descriptive table. (a) Dorsal cephalic schematic chaetotaxy; (b) ventral cephalic schematic chaetotaxy.

**FIGURE 10:**
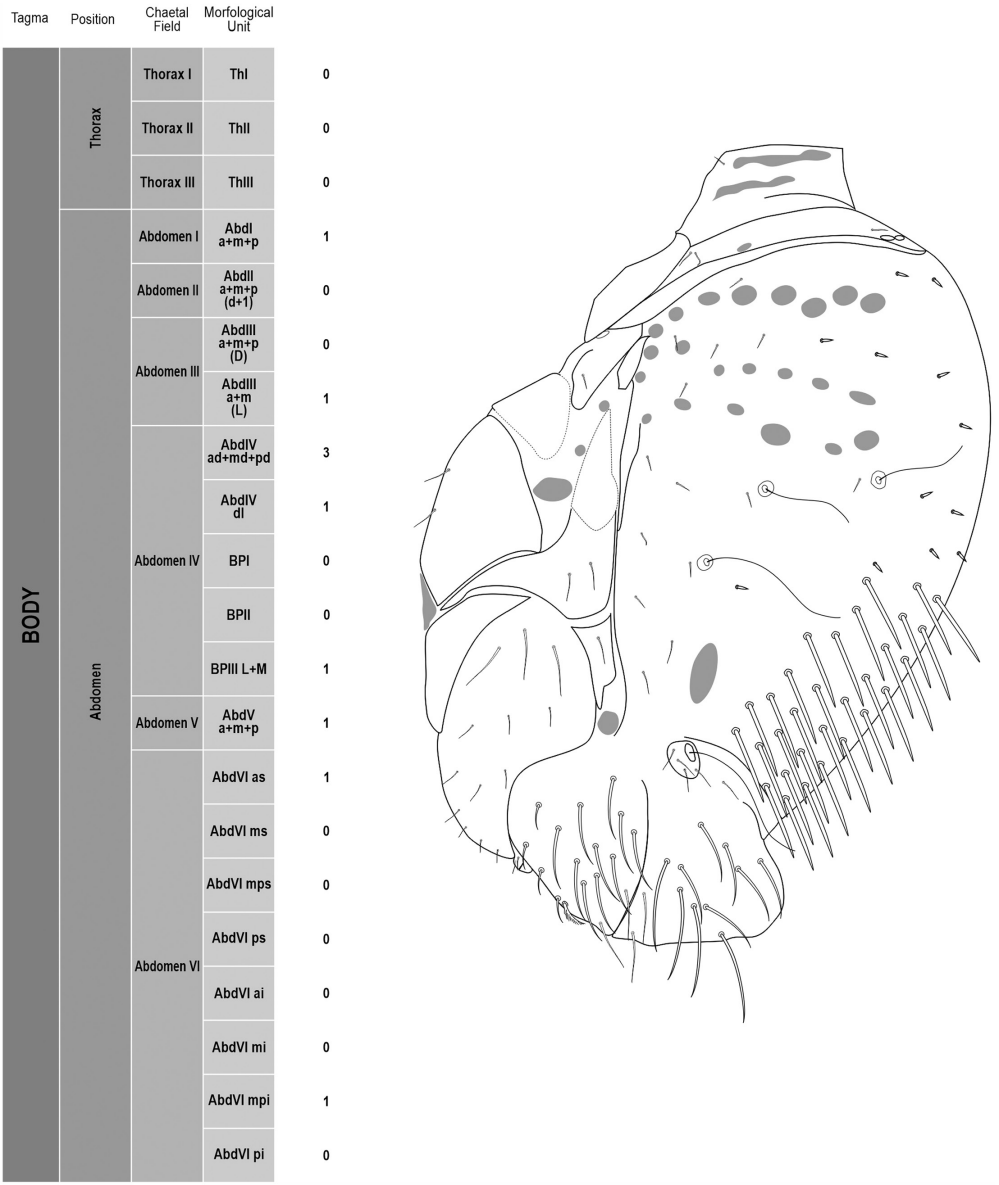
*Pararrhopalites hermesi* **sp. n.** body chaetotaxy and descriptive table.

**FIGURE 11:**
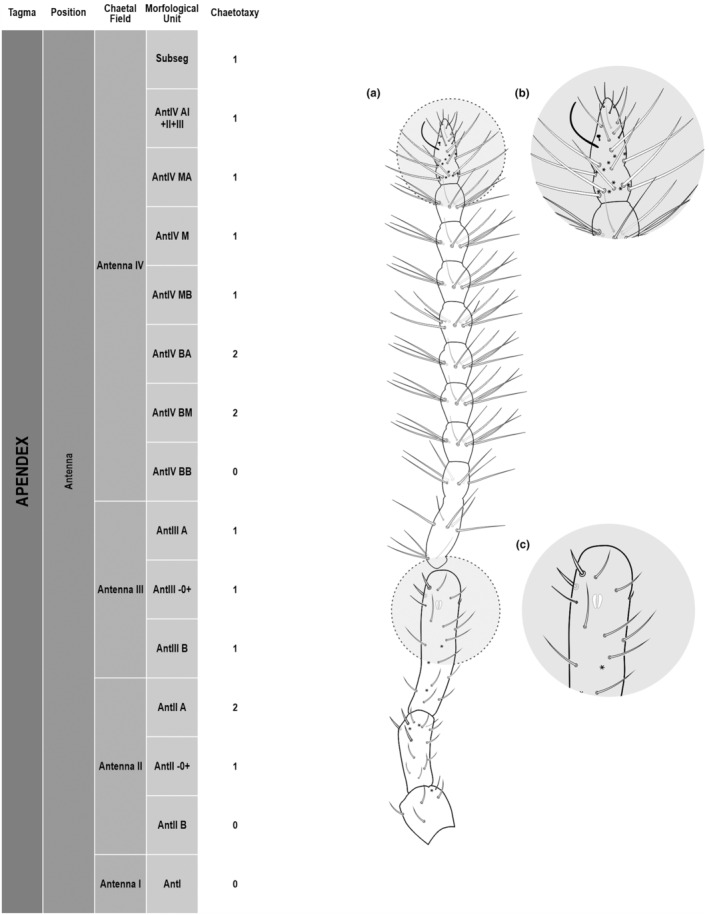
*Pararrhopalites hermesi* **sp. n.** antennal chaetotaxy and descriptive table. (a) Whole antennal chaetotaxy; (b) Ant. IV apical subsegment; (c) apical organ on Ant. III.

**FIGURE 12:**
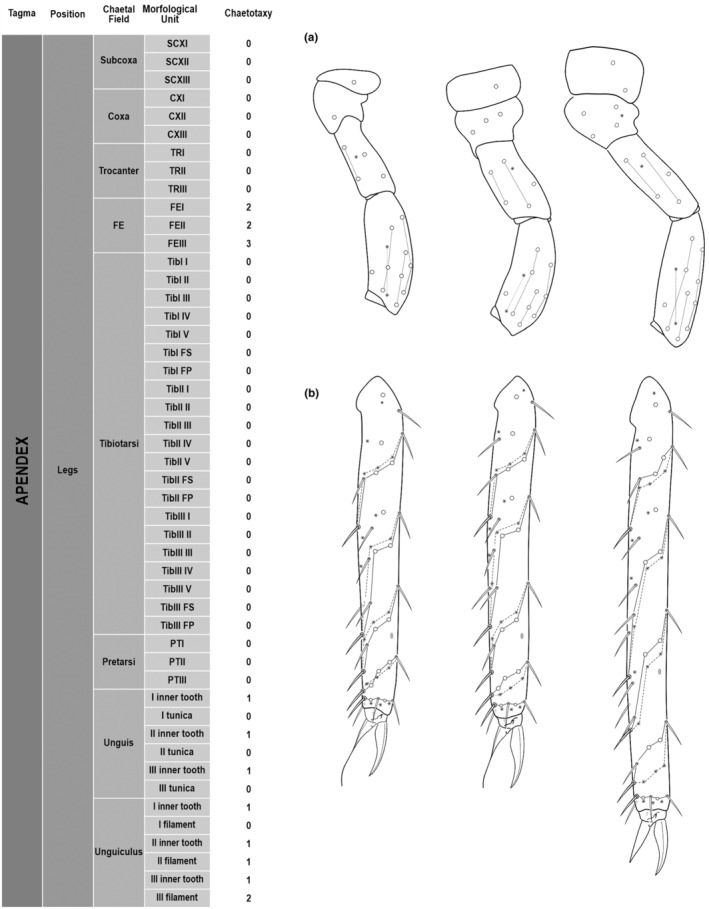
*Pararrhopalites hermesi* **sp. n.** legs chaetotaxy and descriptive table. (a) Subcoxa, coxa, trochanter and femur, legs I–III respectively; (b) tibiotarsus and empodial complex, legs I–III respectively.

**FIGURE 13:**
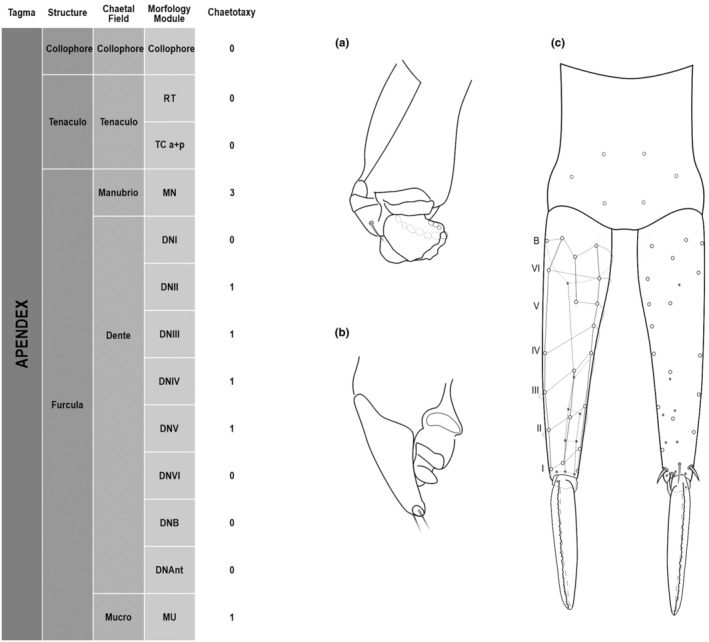
*Pararrhopalites hermesi* **sp. n.** abdominal appendages chaetotaxy. (a) Ventral tube lateral view; (b) tenaculum lateral view; (c) furcula chaetotaxy and mucronal lamellae. Solid circles – anterior view, hollow circles – posterior view.

zoobank.org:act:554455DB‐364D‐4C48‐A0A9‐33CC578C4E02

(Figures 8, 9, 10, 11, 12, 13)

**Type material.** Holotype female in slide (#6040/CRFS/UEPB): Brazil, Minas Gerais state, Nova Lima municipality, cave RM‐33, 20°02′05.30″ S, 43°59′40.20″ W, 30.VI.2014, Bioespeleo et al. coll. Paratype in slide (#6038/CRFS/UEPB): 1 female, Brazil, Minas Gerais State, Nova Lima municipality, RM‐33, 20°02′05.30″ S, 43°59′40.20″ W, 02.IV.2014, Bioespeleo et al. coll. Paratype in slide (#6039/CRFS/UEPB): 1 female, same data as holotype. Paratype in slide (#6042/CRFS/UEPB): 1 male, Brazil, Minas Gerais State, Nova Lima municipality, RM‐03, 20°02′37.50″ S 44°00′22.40″ W, 31.III.2014, Bioespeleo et al. coll. Paratype in slide (#18060/CRFS/UEPB): 1 female, Brazil, Minas Gerais State, Nova Lima municipality, RM‐33, 20°02′05.30″ S, 43°59′40.20″ W, 21.X.2017, A. S. Reis & R. Zampaulo coll.

Additional materials https://doi.org/10.15468/pbxmgz

**Etymology.** *Pararrhopalites hermesi* **sp. n.** is named after the Greek god Hermes, in allusion to the wings of his flying shoes, quite similar in shape to the subanal appendages of this species.

#### 3.3.1 Habitat and distribution

This species is known from caves and SSH in a range over 200 km across different lithologies. It is a regionally widespread SSH species, but it is not abundant, as there are less than 20 records of this species.

#### 3.3.2 Remarks

This species is part of a group with a specific kind of subanal appendages (number 28 in Figure 8), which includes *P. queirozi* and *P. fallaciosus* **sp. n.** The species has an intermediary number of subsegments on Ant. IV (nine subsegments), has no eyes, lacks the corner tooth in all unguiculi, and the inner lamella of the mucro is smooth. This combination of features can easily differentiate the three species.

Despite its wide distribution, *P. hermesi* **sp. n.** presents some features that may be indicative of its relation to the SSH environment, for instance eye reduction, Ant. IV shorter (nine subsegments), overall small body size, and the reduction of the corner tooth and apical filament on unguiculus.

### 3.4 *Pararrhopalites atypicus* sp. n

**FIGURE 14:**
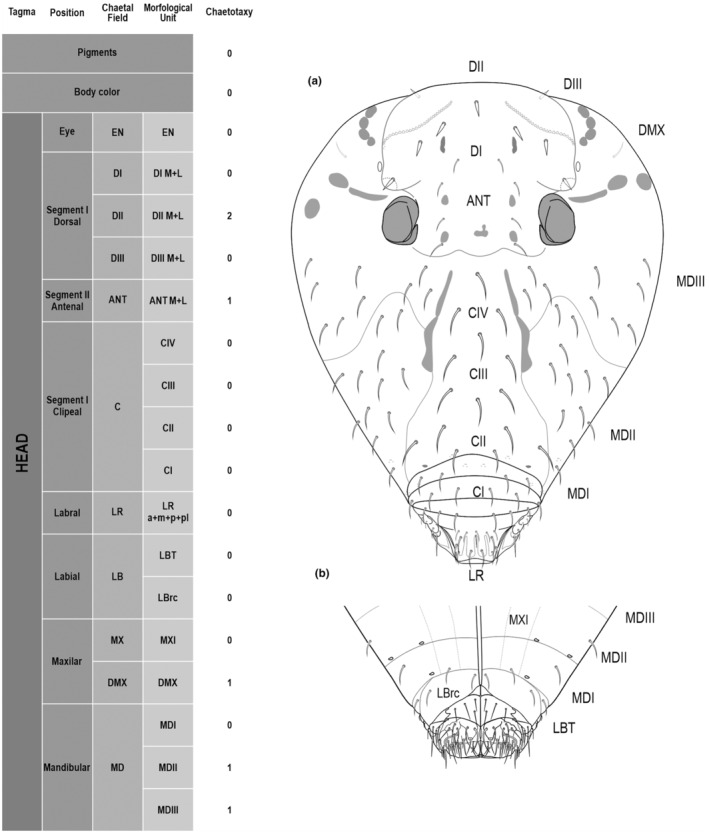
*Pararrhopalites atypicus* **sp. n.** cephalic chaetotaxy and descriptive table. (a) Dorsal cephalic schematic chaetotaxy; (b) ventral cephalic schematic chaetotaxy.

**FIGURE 15:**
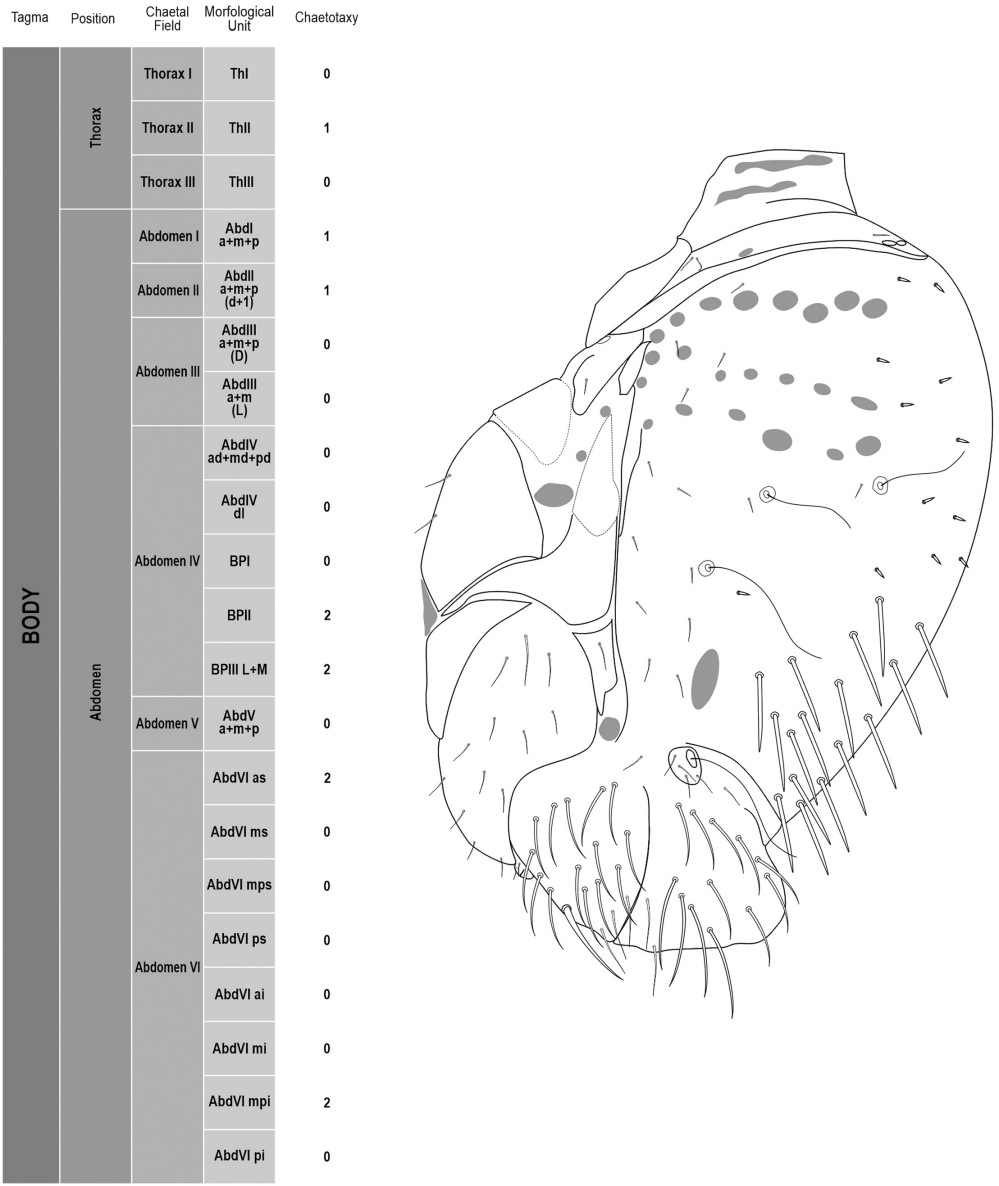
*Pararrhopalites atypicus* **sp. n.** body chaetotaxy and descriptive table.

**FIGURE 16:**
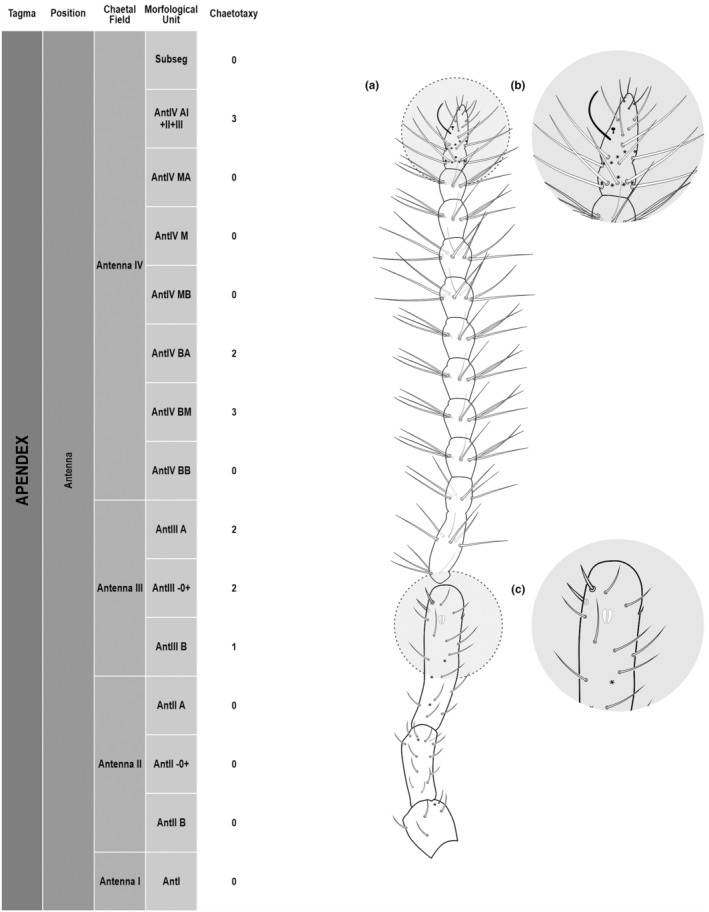
*Pararrhopalites atypicus* **sp. n.** antennal chaetotaxy and descriptive table. (a) Whole antennal chaetotaxy; (b) Ant. IV apical subsegment; (c) apical organ on Ant. III.

**FIGURE 17:**
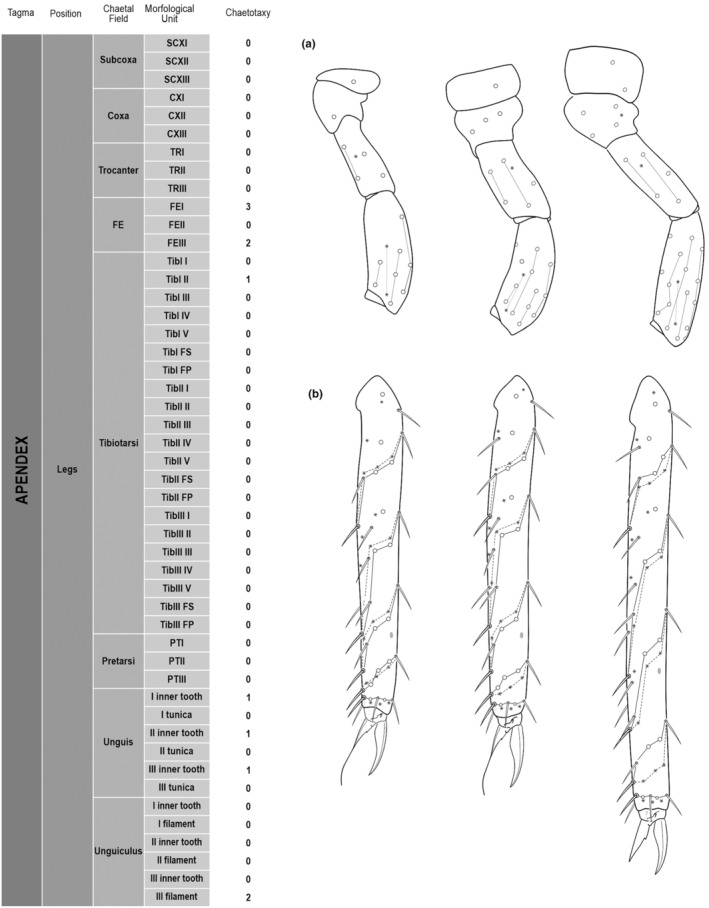
*Pararrhopalites atypicus* **sp. n.** legs chaetotaxy and descriptive table. (a) Subcoxa, coxa, trochanter and femur, legs I–III respectively; (b) tibiotarsus and empodial complex, legs I–III respectively.

**FIGURE 18:**
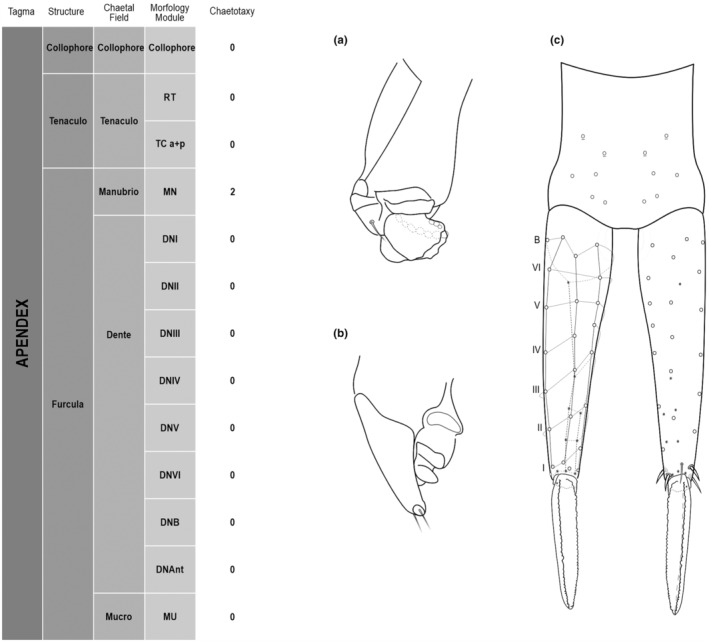
*Pararrhopalites atypicus* **sp. n.** abdominal appendages chaetotaxy. (a) Ventral tube lateral view; (b) tenaculum lateral view; (c) furcula chaetotaxy and mucronal lamellae. Solid circles – anterior view, hollow circles – posterior view.

zoobank.org:act:14BAAE02‐F5FB‐4169‐BCE8‐9AFC6DE4FBB1

(Figures 8 and 14, 15, 16, 17, 18)

**Type material.** Holotype female in slide (#2142/CRFS/UEPB): Brazil, Minas Gerais State, Mariana municipality, cave FN‐05, 20°13′17.81″ S 43°26′02.93″ W, 12.XII.2012, Bioespeleo et al. coll. Paratypes in slides (#2139, #2140, #2141, #2143, #2144/CRFS/UEPB): 4 females and 1 male, same data as holotype.

Additional materials https://doi.org/10.15468/pbxmgz

**Etymology.** *P. atypicus* **sp. n.** is Latin for unusual, as this is the only species of this region which lacks the interantennal sensillar triangle, known so far.

#### 3.4.1 Habitat and distribution

This species is restricted to a single area, there are only 10 records for three small caves in the same iron rock formation. The species is most likely distributed along the *canga*, a SSH formation resulting of weathering of the iron rock, that often connect caves in the same lithology.

#### 3.4.2 Remarks

This species resembles *P. sideroicus* Zeppelini & Brito, 2014 and *P. ubiquum* Zeppelini et al., 2018, in the shape of the subanal appendages and general body chaetotaxy but differ from all the species of the genus with records in this area by lacking the interantennal sensillar triangle, this feature seems to be shared by both the species of the queirozi‐group and the ubiquum‐group. The presence of only three chaetae in the dorsal cephalic area DII is also very unusual for the genus and the reduced number of chaetae in the dorsal posterior part of the great abdomen can be diagnostic for this species.

### 3.5 *Pararrhopalites ritaleeae* sp. n

**FIGURE 19:**
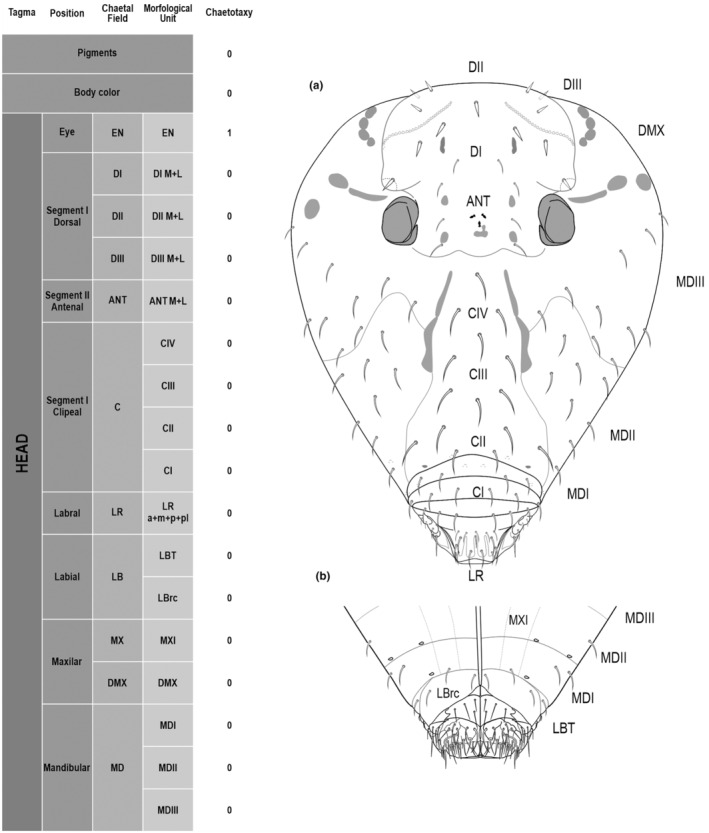
*Pararrhopalites ritaleeae* **sp. n.** cephalic chaetotaxy and descriptive table. (a) Dorsal cephalic schematic chaetotaxy; (b) ventral cephalic schematic chaetotaxy.

**FIGURE 20:**
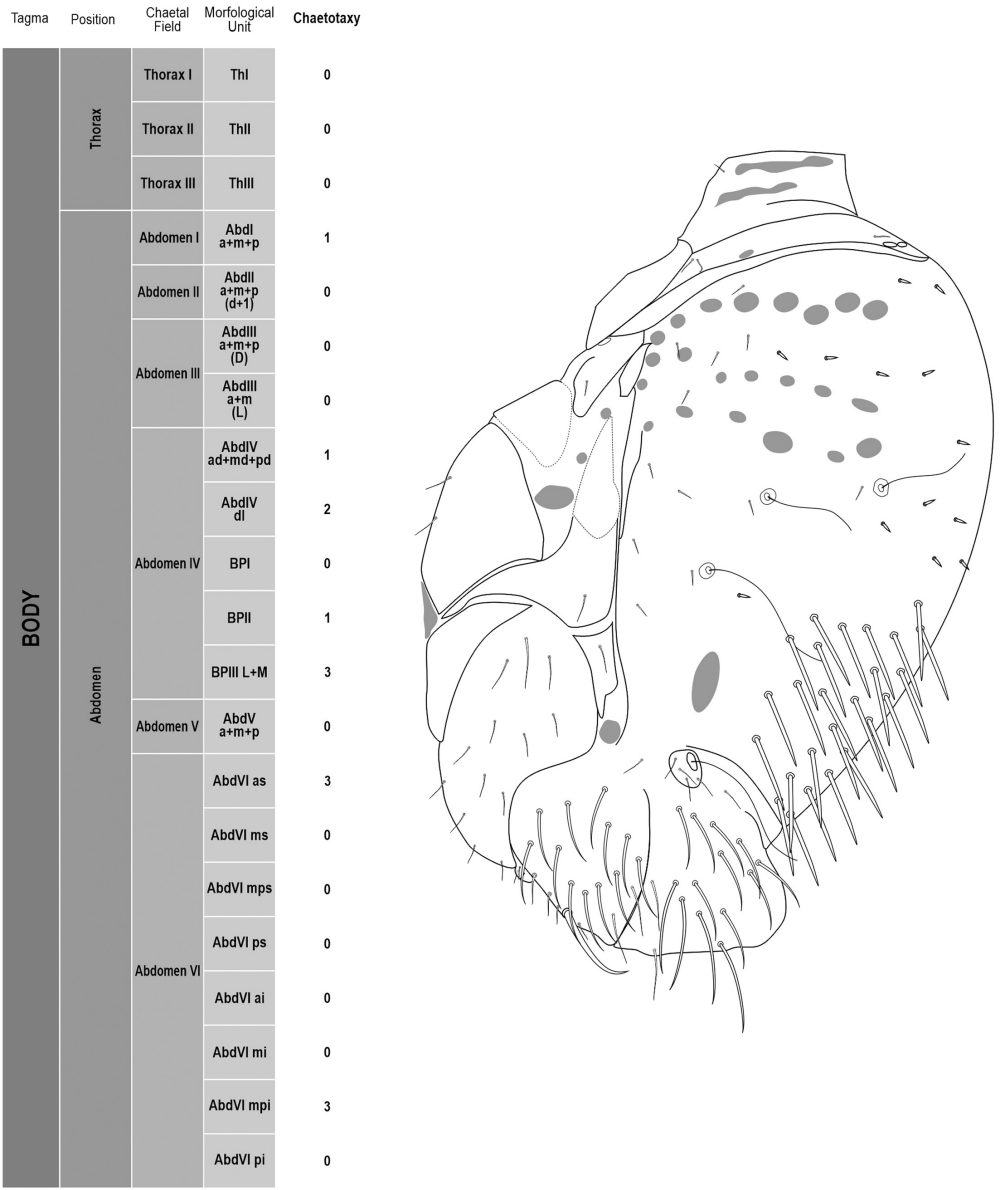
*Pararrhopalites ritaleeae* **sp. n.** body chaetotaxy and descriptive table.

**FIGURE 21:**
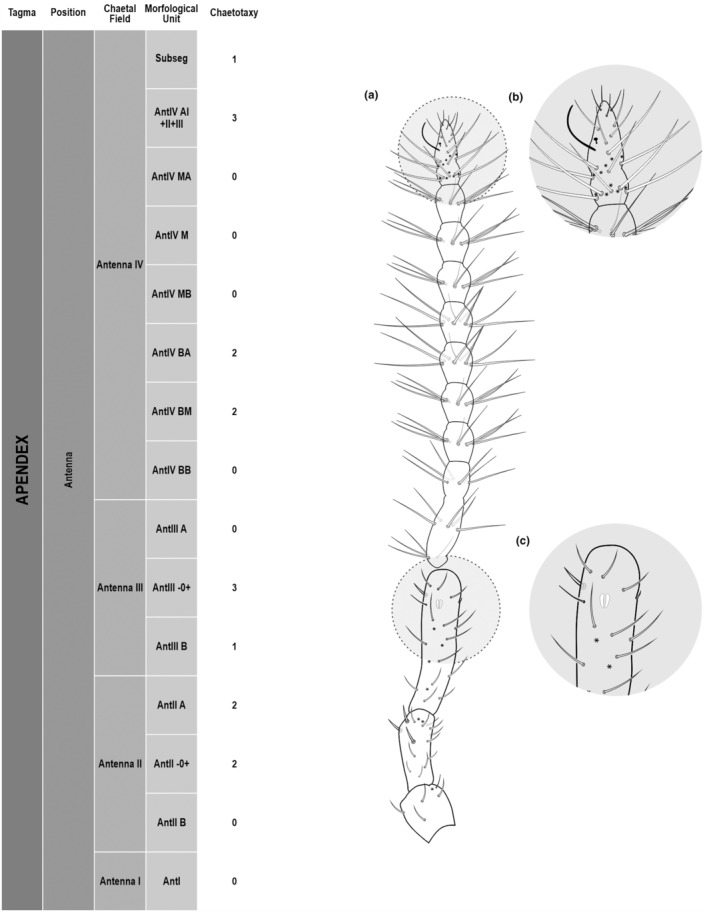
*Pararrhopalites ritaleeae* **sp. n.** antennal chaetotaxy and descriptive table. (a) Whole antennal chaetotaxy; (b) Ant. IV apical subsegment; (c) apical organ on Ant. III.

**FIGURE 22:**
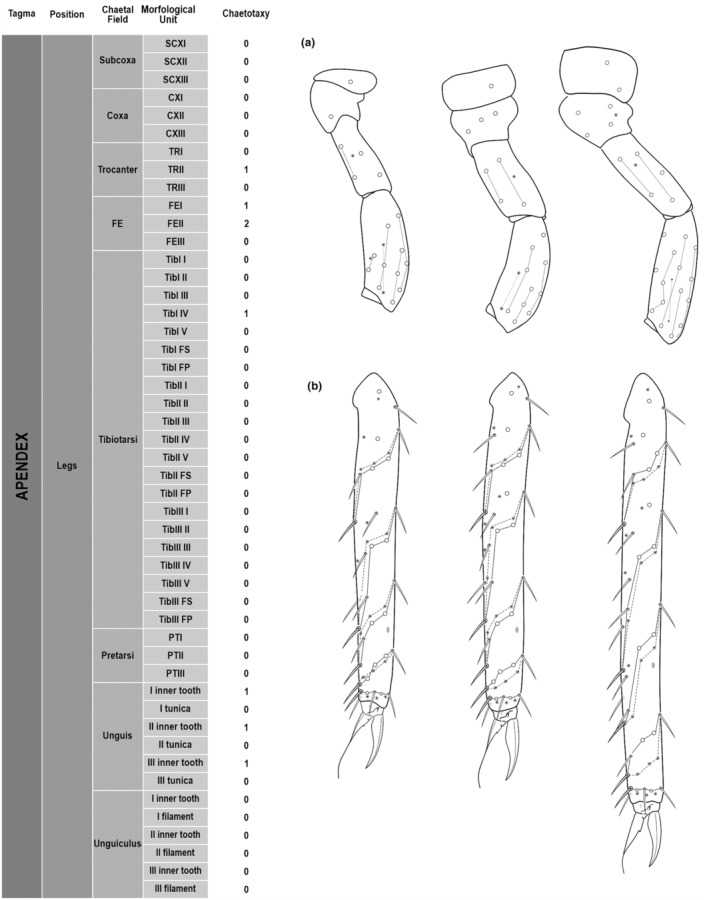
*Pararrhopalites ritaleeae* **sp. n.** legs chaetotaxy and descriptive table. (a) Subcoxa, coxa, trochanter and femur, legs I–III respectively; (b) tibiotarsus and empodial complex, legs I–III respectively.

**FIGURE 23:**
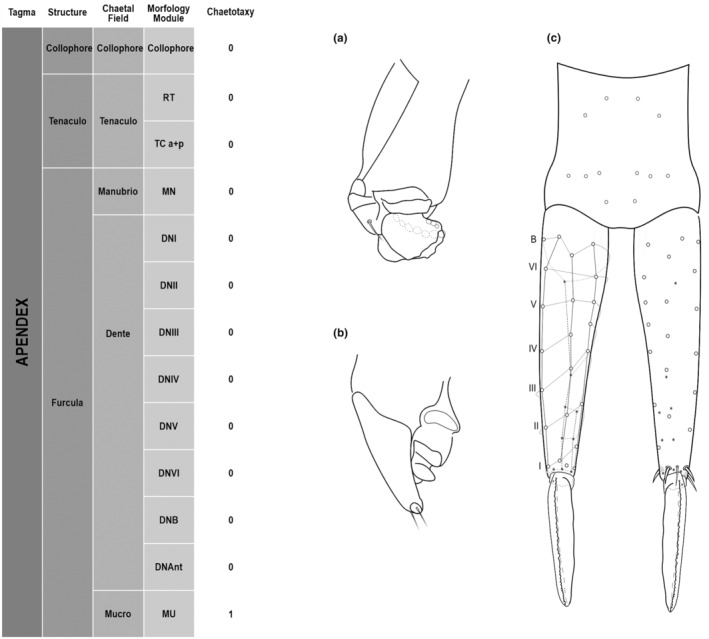
*Pararrhopalites ritaleeae* **sp. n.** abdominal appendages chaetotaxy. (a) Ventral tube lateral view; (b) tenaculum lateral view; (c) furcula chaetotaxy and mucronal lamellae. Solid circles – anterior view, hollow circles – posterior view.

zoobank.org:act:C531BF30–7489‐449F‐B0A1‐2B4F1BBF67EF

(Figures 8 and 19, 20, 21, 22, 23)

**Type material.** Holotype female in slide (#4942/CRFS/UEPB): Brazil, Minas Gerais state, Itabirito municipality, cave VL‐35, 20°18′28.30″ S, 43°56′32.30″ W, 18.VI.2013, Mascarenhas et al. coll. Paratypes in slides (#2577, #2587, #2595/CRFS/UEPB): 3 females, Brazil, Minas Gerais state, Itabirito municipality, cave VL‐35, 20°18′28.30″ S, 43°56′32.30″ W, 07‐10.V.2013, Mascarenhas et al. coll. Paratypes in slides (#4938, #4940, #4943/CRFS/UEPB): 2 female and 1 male, same data as holotype.

Additional materials https://doi.org/10.15468/pbxmgz

**Etymology.** *Pararrhopalites ritaleeae* **sp. n.** is named in memoriam after Rita Lee a Brazilian singer deceased may, 2023.

#### 3.5.1 Habitat and distribution

*Pararrhopalites ritaleeae* **sp. n.** is the most abundant species presented here, but its distribution is restricted to a group of seven caves in the same iron rock formation in the type locality. It is known only from caves, but surface samplings are needed to survey the epigeic fauna.

#### 3.5.2 Remarks

This species is also part of the ubiquum‐group and resembles the other species of the group by the shape of the subanal appendages and general body chaetotaxy. The absence of eyes is observed in *P. ubiquum*, and the apical filament of the unguiculus III exceeding the tip of the unguis is similar to *P. sideroicus*. The features that differentiate *P. ritaleeae* **sp. n.** are the mucro inner lamella smooth, Ant IV with nine subsegments, unguiculus III apical filament exceeding unguis tip, Abd. IV lateral chaetae lacking.

### 3.6 *Pararrhopalites ironicus* sp. n

**FIGURE 24:**
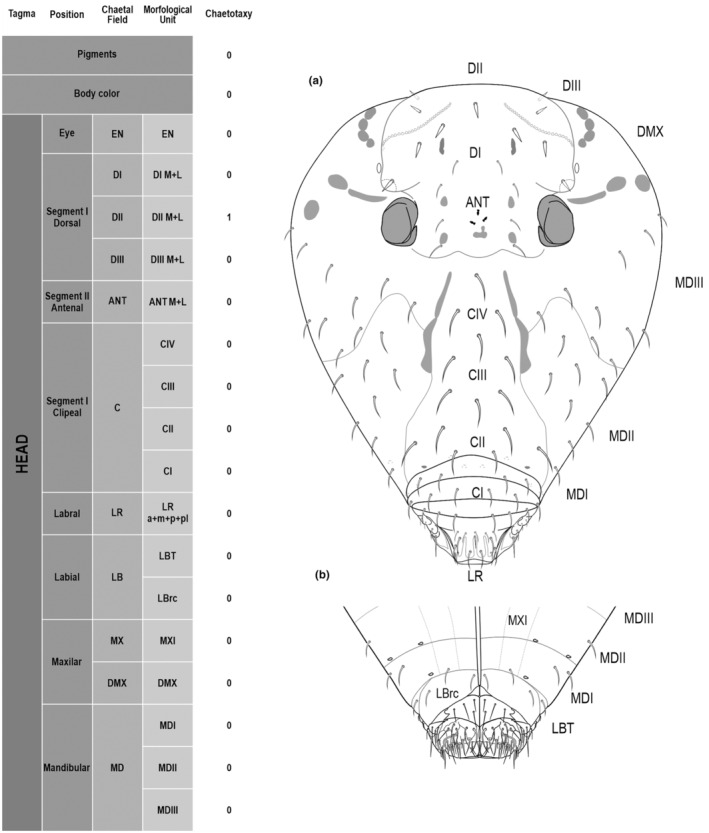
*Pararrhopalites ironicus* **sp. n.** cephalic chaetotaxy and descriptive table. (a) Dorsal cephalic schematic chaetotaxy; (b) ventral cephalic schematic chaetotaxy.

**FIGURE 25:**
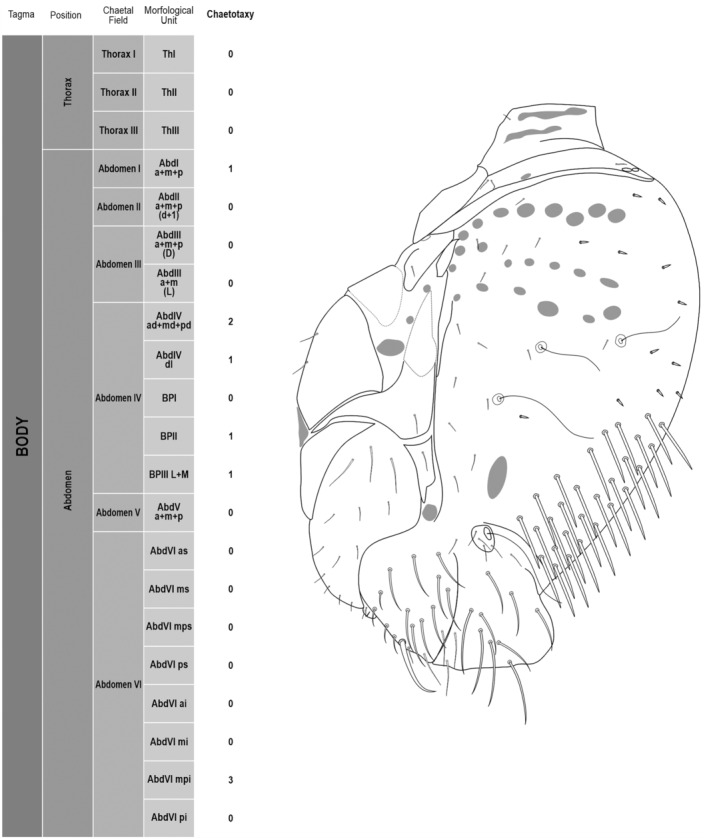
*Pararrhopalites ironicus* **sp. n.** body chaetotaxy and descriptive table.

**FIGURE 26:**
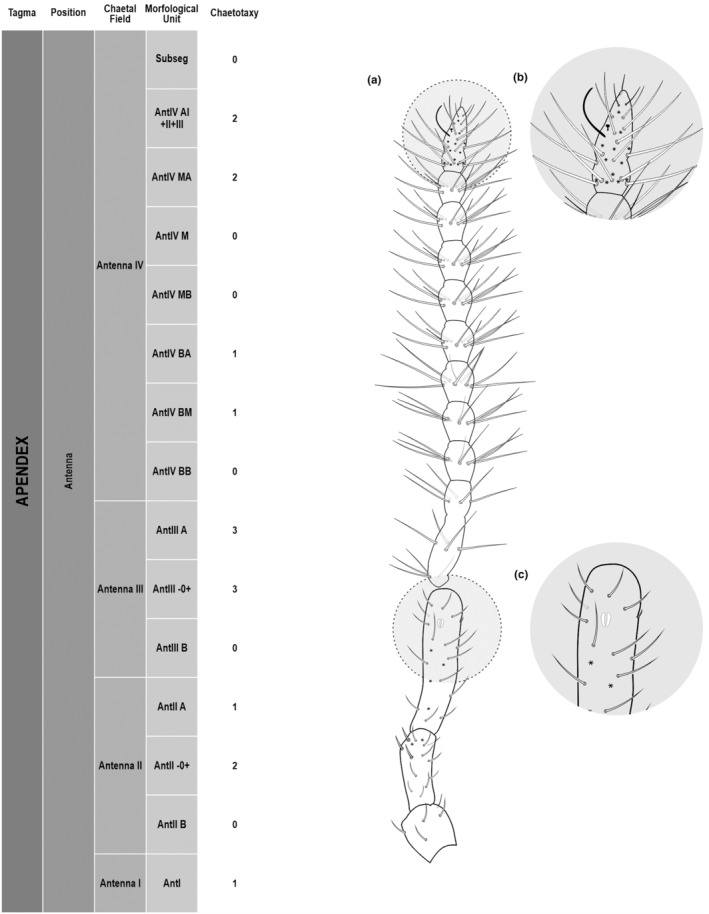
*Pararrhopalites ironicus* **sp. n.** antennal chaetotaxy and descriptive table. (a) Whole antennal chaetotaxy; (b) Ant. IV apical subsegment; (c) apical organ on Ant. III.

**FIGURE 27:**
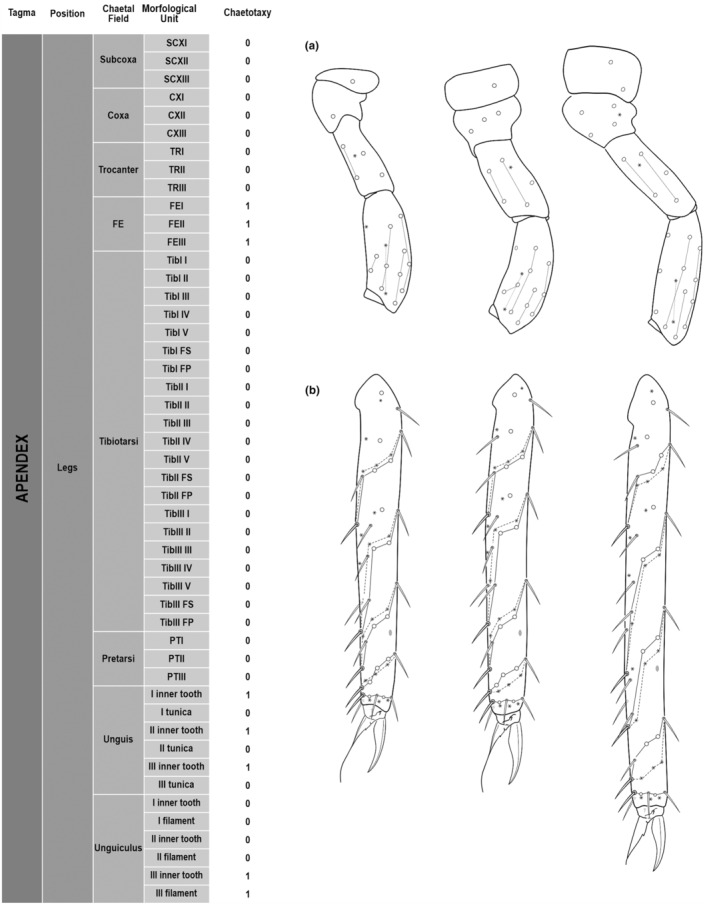
*Pararrhopalites ironicus* **sp. n.** legs chaetotaxy and descriptive table. (a) Subcoxa, coxa, trochanter and femur, legs I–III respectively; (b) tibiotarsus and empodial complex, legs I–III respectively.

**FIGURE 28:**
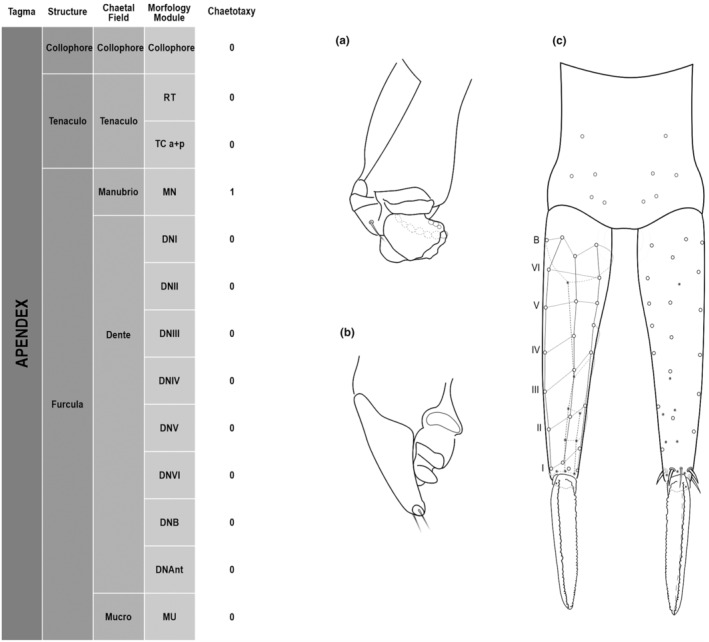
*Pararrhopalites ironicus* **sp. n.** abdominal appendages chaetotaxy. (a) Ventral tube lateral view; (b) tenaculum lateral view; (c) furcula chaetotaxy and mucronal lamellae. Solid circles – anterior view, hollow circles – posterior view.

zoobank.org:act:47BFB22F‐967A‐437D‐A9FA‐63F1675FB00B

(Figures 8 and 24, 25, 26, 27, 28)

**Type material.** Holotype female in slide (#18545/CRFS/UEPB): Brazil, Minas Gerais state, Barão dos Cocais municipality, cave BRU‐0028, 19°52′49.00″ S, 43°26′13.50″ W, 20‐24.IX.2021, Spelayon et al. coll. Paratypes in slides (#18555, #18566/CRFS/UEPB): 2 females, same data as holotype. Paratypes in slides (#19047, #19048, #19059/CRFS/UEPB): 2 females and 1 male, Brazil, Minas Gerais state, Barão dos Cocais municipality, cave MDIR‐0028, 19°52′49.00″ S, 43°26′13.50″ W, 14‐18.III.2022, Spelayon et al. coll.

No additional materials available.

**Etymology.** *Pararrhopalites ironicus* **sp. n.**, from iron and the Greek suffix “*icus*” (belonging or related to), in allusion to its relation to the iron rock.

#### 3.6.1 Habitat and distribution

*Pararrhopalites ironicus* **sp. n.** is a very restricted species, represented by only six records from two caves in the same iron rock formation, called *Serra do Tamanduá*, the caves are 2700 m away from each other, and connected by SSH. It is a rare species, likely to be confined to the subterranean environment.

#### 3.6.2 Remarks

This is one more species of the ubiquum‐group, this species is very similar to *P. sideroicus*, *P. ubiquum*, *P. atypicus* **sp. n.**, and *P. ritaleeae* **sp. n.** in the general body chaetotaxy. The main differences are to be found in the dorsal cephalic chaetotaxy. *Pararrhopalites ironicus* **sp. n.** can be differentiated from those species by presenting only five spines (type 8 in Figure 8), the Ant. I with Only five chaetae, the apical organ of Ant. III chaeta ape is a normal chaeta (type 21 in Figure 8), unguiculus III apical filament reaching but not exceeding the tip of the unguis and lacking the corner tooth.

## 4 DISCUSSION

To access the global species diversity, it is mandatory to enhance the description rates of new taxa, mainly where the biodiversity is least known. A description protocol that can communicate the morphologic characters in a coded notation, allows the application of new technologies in the research and machine learning, which may be a major turnover in the discipline, and affect the species description rates. The scarcity of trained taxonomists and the hermeticity of the taxonomic description manuscripts are the biggest barriers to the advance of the knowledge on the species diversity and evolutionary processes of diversification, both clue elements to understand the global biodiversity decline.

In the study of Collembola, as well as in many other groups, the information content of a traditional taxonomic text is often difficult to access and cannot be transported to analytical software without a detailed revision of the species description, which many times demands an expert in the taxon. The traditional format is also almost impossible to be used for machine learning, as there are many differences in the presentation of the data, that can make the comparison among different manuscripts impossible to non‐experts and to artificial intelligence. The open character list of the coded description allows easy insertion and correction of the information, and the character lists, the chaetae banks and the coded species descriptions are fully compatible with technologies that work with data matrices.

Our results can be synthesized in the following conclusions:

1. The coded taxonomic description is a notation method that produces interchangeable data, fully available for different scientific disciplines. The data can be used by non‐specialists for different purposes in science.
2. The method makes it possible to add any source of new data to the description when it becames available. It is dynamic and open as a continuous list of characters, the updating of the knowledge of a given species is not dependent of a traditional taxonomic revision.
3. The method allows machine learning that can help to speed the taxon identification and species description rates where they are least known. This can be an important tool to fight global biodiversity crisis.
4. Coded description is idealized to Collembola but must be applied to any taxonomic group, reducing the ambiguity of narrative descriptions. Once it is widely used, the comparative analysis will be almost a straightforward process.

## AUTHOR CONTRIBUTIONS

**Douglas Zeppelini:** Conceptualization (equal); formal analysis (equal); funding acquisition (equal); investigation (equal); methodology (equal); project administration (equal); supervision (equal); writing – original draft (equal); writing – review and editing (equal). **Misael Augusto de Oliveira‐Neto:** Conceptualization (equal); investigation (equal); methodology (equal); software (equal); visualization (equal). **João Victor Lemos Cavalcante de Oliveira:** Data curation (equal); formal analysis (equal); investigation (equal); methodology (equal); visualization (equal); writing – review and editing (equal). **Aila Soares Ferreira:** Data curation (equal); formal analysis (equal); investigation (equal); methodology (equal); visualization (equal); writing – review and editing (equal). **Roniere Andrade de Brito:** Data curation (equal); formal analysis (equal); investigation (equal); methodology (equal); validation (equal); visualization (equal); writing – review and editing (equal). **Bruna Carolline Honório Lopes:** Data curation (equal); investigation (equal); visualization (equal). **Nathan Paiva Brito:** Data curation (equal); investigation (equal); visualization (equal). **Luis Carlos Stievano:** Methodology (equal); visualization (equal). **Estevam Cipriano Araujo de Lima:** Conceptualization (equal); formal analysis (equal); investigation (equal); methodology (equal); visualization (equal); writing – review and editing (equal).

## CONFLICT OF INTEREST STATEMENT

The authors declare no conflicts of interest.

## Supporting information


Data S1

## Data Availability

The data that support the findings of this study are openly available in Coleção de Referência de Fauna de Solo at https://www.gbif.org/dataset/8cf92f87‐eaee‐4d1c‐96a9‐49c3f5998351, and in the Data S1.

## 1

The following list of characters is presented in a sequence from the first cephalic segment to the sixth abdominal segment. It is a perfect match of Table 1 and is also represented by an individualized table in each species description plate. This list presents the observed conditions of each character included in our study, a total of 115 morphological characters are listed.

### Color pattern

Pigments:

0 – Absent

1 – Present

Body color:

0 – White

1 – Pale yellow

### Head

Eye number:

0 – 1+1

1 – 0+0

2 – 2+2

#### Dorsal cephalic chaetotaxy

Area DI total chaetae M + L:

0 – 5+5 4(9)6(8)

1 – Different combination.

Area DII total chaetae M + L:

0 – 3+1+3 7(8)

1 – 2+1+2 5(8)

2 – 1+1+1 3(8)

Area DIII total chaetae M + L:

0 – 0+0

1 – Different combination.

#### Antennal area

Antennal area chaetotaxy M + L:

0 – 3+1+3 4(9)3(7)

1 – 2+2 4(9)

#### Clypeal chaetotaxy

Clypeal area CI:

0 – 3+3 6(10)

1 – Different combination.

Clypeal area CII:

0 – 5+1+5 11(10)

1 – Different combination.

Clypeal area CIII:

0 – 2+1+2 5(10)

1 – Different combination.

Clypeal area CIV:

0 – 1+1+1 3(10)

1 – Different combination

#### Labral chaetotaxy

Labral formula a + m + p + pl:

0 – 4+5+5+6 20(10)

1 – Different combination.

#### Mandibular segment chaetotaxy

Area MDI (dorsal + ventral):

0 – 2+2+1+1ov 4(10)2(11)

1 – Different combination.

Area MDII (d + v):

0 – 8+8+2+2ov 16(10)4(11)

1 – 12+12 2+2ov 24(10)4(11)

Area MDIII (d + v):

0 – 10+10 20(10)

1 – 9+9 18(10)

#### Maxilar segment chaetotaxy

Area DMX and

0 – 0+0

1 – 1+1 2(10)

Area MXI

0 – 1+1 4(10)

#### Labial segment chaetotaxy

Labial triangle total chaetae:

0 – 4+4 8(10)

1 – Different combination

Labial remaining chaetae:

0 – 2+2 4(10)

1 – Different combination

### Body

#### Thoracic chaetotaxy

Thorax I total chaetae:

0 – 0+0

1 – at least 1+1

Thorax II total chaetae:

0 – 1+1 2(24)

1 – 0+0

2 – Different combination

Thorax III total chaetae:

0 – 3+3 2(24)2(26)2(23)

1 – Different combination

#### Abdominal chaetotaxy

Abdomen I a + m + p total chaetal:

0 – 3+3 6(25)

1 – 3+3 4(25)2(26)

Abdomen II a + m + p (d + l) total chaetae:

0 – 8+8 8(25)8(26)

1 – 7+7 8(25)6(26)

Abdomen III a + m + p (dorsal) total chaetae:

0 – 10+10 8(25)6(26)6(31)

1 – Different combination

Abdomen III a + m (lateral) total chaetae:

0 – 5+5 10(26)

1 – 5+5 8(26)2(23)

Abdomen IV ad + md + pd total chaetae (dorsal):

0 – 17+17 4(25)30(27)

1 – 27+27 4(25)50(27)

2 – 28+28 56(27)

3 – 30+30 60(27)

Abdomen IV total chaetae (lateral):

0 – 3+3 6(26)

1 – 2+2 4(26)

2 – 0+0

Abdomen IV area BPI total chaetae:

0 – 2+2 4(26)

1 – Different combination

Abdomen IV area BPII total chaetae:

0 – 2+2 4(26)

1 – 1+1 2(26)

2 – 0+0

Abdomen area BPIII L + M total chaetae:

0 – 13+13 10(26)16(18)

1 – 13+13 6(26)20(18)

2 – 13+13 6(17)16(26)4(18)

Abdomen V a + m + p total chaetae:

0 – 7+7 12(26)2(31)

1 – 6+6 10(26)2(31)

Abdomen VI **as** total chaetae:

0 – 3+3 6(26)

1 – 4+4 8(26)

2 – 6+6 12(26)

3 – 7+7 14 (26)

Abdomen VI **ms** total chaetae:

0 – 2+2 4(26)

1 – Different combination

Abdomen VI **mps** total chaetae:

0 – 3+1+3 7(30)

1 – Different combination

Abdomen VI **ps** total chaetae:

0 – 1+1+1 3(26)

1 – Different combination

Abdomen VI **aai** + **ai** total chaetae:

0 – 8+8 16(26)

1 – Different combination

Abdomen VI **mi** total chaetae:

0 – 3+3 6(26)

1 – Different combination

Abdomen VI **mpi** total chaetae:

0 – 4+4 6(26)2(28)

1 – 5+5 8(26)2(28)

2 – 4+4 6(26)2(29)

3 – 5+5 8(26)2(29)

Abdomen VI **pi** total chaetae:

0 – 3+3 6(26)

1 – Different combination.

### Appendages

#### Antenna

Antenna I total chaetae:

0 – 6 5(9)1(21)

1 – 5 4(9)1(21)

Antenna II whorl **A** total chaetae:

0 – 7 6(9)1(21)

1 – 7 2(20)1(21)4(9)

2 – 8 2(20)1(21)5(9)

Antenna II whorls −, **0** and + total chaetae:

0 – 3 3(9)

1 – 3 1(20)2(9)

2 – 4 1(20)3(9)

Antenna II whorl **B** total chaetae:

0 – 4 4(9)

1 – Different combination

Antenna III whorl **A** total chaetae:

0 – 8 2(6)1(2)1(21)1(20)3(9)

1 – 8 2(6)1(2)1(21)2(20)2(9)

2 – 8 2(6)1(2)2(20)3(9)

3 – 8 2(6)1(2)5(9)

Antenna III whorls −, **0** and + total chaetae:

0 – 8 4(9)4(5)

1 – 9 1(9)8(10)

2 – 9 9(10)

3 – 10 10(10)

Antenna III whorl **B** total chaetae:

0 – 4 4(9)

1 – 5 5(9)

Antenna IV subsegments:

0 – 10 subsegments

1 – 9 subsegments

2 – Different combination.

Antenna IV **AI** + **AII** + **AIII** total chaetae:

0 – 34 1(3)1(1)2(4)6(10)24(5)

1 – 32 1(3)1(1)2(4)8(10)20(5)

2 – 31 1(3)1(1)5(10)24(5)

3 – 30 1(3)1(1)8(10)20(5)

Antenna IV **MA** total chaetae per whorl:

0 – 8 1(4)7(5)

1 – 9 1(4)8(5)

2 – 10 2(4)8(5)

Antenna IV **M** total chaetae per whorl:

0 – 9 1(4)8(5)

1 – 10 2(4)8(5)

Antenna IV **MB** total chaetae per whorl:

0 – 8 1(4)7(5)

1 – 9 1(4)8(5)

Antenna IV **BA** total chaetae:

0 – 3 3(5)

1 – 5 5(5)

2 – 6 6(5)

Antenna IV **BM** total chaetae:

0 – 3 3(5)

1 – 4 4(5)

2 – 5 5(5)

3 – 6 5(5)1(4)

Antenna IV **BB** total chaetae:

0 – 4 4(5)

1 – Different combination

#### Legs

Subcoxa I total chaetae:

0 – 1 1(26)

1 – Different combination

Subcoxa II total chaetae:

0 – 1 1(26)

1 – Different combination

Subcoxa III total chaetae:

0 – 2 2(26)

1 – Different combination

Coxa I total chaetae:

0 – 1 1(26)

1 – Different combination

Coxa II total chaetae:

0 – 3 3(26)

1 – Different combination

Coxa III total chaetae:

0 – 5 5(26)

1 – Different combination

Trochanter I total chaetae:

0 – 5 5(26)

1 – Different combination

Trochanter II total chaetae:

0 – 6 6(26)

1 – 5 5(26)

Trochanter III total chaetae:

0 – 6 1(15)5(26)

1 – Different combination

Femur I total chaetae:

0 – 15 15(26)

1 – 14 14(26)

2 – 12 12(26)

3 – 10 10(26)

Femur II total chaetae:

0 – 15 13(26)2(13)

1 – 14 14(26)

2 – 12 12(26)

Femur III total chaetae:

0 – 18 18(26)

1 – 14 13(26)1(21)

2 – 14 14(26)

3 – 12 12(26)

Tibiotarsus I whorl I total chaetae:

0 – 9 1(13)8(14)

1 – Different combination

Tibiotarsus I whorl II total chaetae:

0 – 8 1(13)7(16)

1 – 9 1(13)8(16)

Tibiotarsus I whorl III total chaetae:

0 – 8 1(13)7(16)

1 – Different combination

Tibiotarsus I whorl IV total chaeta:

0 – 14 1(13)13(16)

1 – 12 1(13)11(16)

2 – Different combination

Tibiotarsus I whorl V total chaetae:

0 – 8 1(13)7(16)

1 – Different combination

Tibiotarsus I **FS** area total chaetae:

0 – 2 2(16)

1 – Different combination

Tibiotarsus I **FP** area total chaetae:

0 – 3 3(16)

1 – Different combination

Tibiotarsus II whorl I total chaetae:

0 – 9 1(13)8(14)

1 – Different combination

Tibiotarsus II whorl II total chaetae:

0 – 8 1(13)7(16)

1 – Different combination

Tibiotarsus II whorl III total chaetae:

0 – 8 1(13)7(16)

1 – Different combination

Tibiotarsus II whorl IV total chaetae:

0 – 14 1(13)13(16)

1 – Different combination

Tibiotarsus II whorl V total chaeta:

0 – 8 1(13)7(16)

1 – Different combination

Tibiotarsus II **FS** area total chaetae:

0 – 3 3(16)

1 – Different combination

Tibiotarsus II **FP** area total chaetae:

0 – 3 3(16)

1 – Different combination

Tibiotarsus III whorl I total chaetae:

0 – 9 1(13)8(14)

1 – Different combination

Tibiotarsus III whorl II total chaetae:

0 – 8 1(13)7(16)

1 – Different combination

Tibiotarsus III whorl III total chaetae:

0 – 8 1(13)7(16)

1 – Different combination

Tibiotarsus III whorl IV total chaetae:

0 – 14 2(13)12(16)

1 – Different combination

Tibiotarsus III whorl V total chaetae:

0 – 8 1(13)7(16)

1 – Different combination

Tibiotarsus III **FS** area total chaetae:

0 – 3 3(16)

1 – Different combination

Tibiotarsus III **FP** area total chaetae:

0 – 3 3(16)

1 – Different combination

Pretarsus I pre tarsal chaetae:

0 – 1+1 2(12)

1 – Different combination

Pretarsus II pre tarsal chaetae:

0 – 1+1 2(12)

1 – Different combination

Pretarsus III pre tarsal chaetae:

0 – 1+1 2(12)

1 – Different combination

Unguis I inner tooth:

0 – present

1 – absent

Unguis I tunica:

0 – absent

1 – present

Unguis II inner tooth:

0 – present

1 – absent

Unguis II tunica:

0 – absent

1 – present

Unguis III inner tooth:

0 – present

1 – absent

Unguis III tunica:

0 – absent

1 – present

Unguiculus I inner tooth:

0 – present

1 – absent

Unguiculus I apical filament:

0 – exceeding the unguis tip

1 – reaching the unguis tip

Unguiculus II inner tooth:

0 – present

1 – absent

Unguiculus II apical filament:

0 – exceeding the unguis tip

1 – reaching the unguis tip

Unguiculus III inner tooth:

0 – present

1 – absent

Unguiculus III apical filament:

0 – exceeding the unguis tip

1 – reaching the unguis tip

2 – shorter than unguis tip

Collophore total chaetae:

0 – 1+1 2(19)

1 – different combination

Tenaculum rami:

0 – 3+3 teeth

1 – different combination

Tenaculum Corpus (a + p):

0 – 2 2(18)

1 – different combination

Manubrium total chaetae:

0 – 6+6 12(16)

1 – 5+5 10(16)

2 – 4+4 8(16)

3 – 3+3 4(16)2(22)

Dental whorl I total chaetae:

0 – 5 1(16)4(22)

1 – different combination

Dental whorl II total chaetae:

0 – 3 3(16)

1 – 3 1(16)2(22)

Dental whorl III total chaetae:

0 – 3 3(16)

1 – 3 1(16)2(22)

Dental whorl IV total chaetae:

0 – 3 3(16)

1 – 2 2(22)

Dental whorl V total chaetae:

0 – 3 3(16)

1 – 2 1(16)1(22)

Dental whorl VI total chaetae:

0 – 2 2(16)

1 – different combination

Dental basal area **B** total chaetae:

0 – 4 4(16)

1 – different combination

Dental anterior chaetotaxy:

0 – 4,2,2,1,1

1 – different combination

Mucro lamellae:

0 – Both lamellae serrated

1 – Internal lamella smooth

## References

[ece311206-bib-0001] Bardgett, R. D. , & van der Putten, W. H. (2014). Belowground biodiversity and ecosystem functioning. Nature, 515, 505–511. 25428498 10.1038/nature13855

[ece311206-bib-0002] Bellinger, P. F. , Christiansen, K. A. , & Janssens, F. (1996). Checklist of the Collembola of the world . Retrieved June 20, 2023, from http://www.collembola.org

[ece311206-bib-0003] Betsch, J.‐M. (1980). Éléments pour une Monographie des Collemboles Symphyplêones (Hexapodes, Aptérygotes). Mémoires du Muséum National D'Histoire Naturelle.

[ece311206-bib-0004] Betsch, J.‐M. (1997). An ontogenetically focused chaetotaxical scheme in Symphypleona (Collembola): The 6th abdominal segment. Pedobiologia, 41, 13–18.

[ece311206-bib-0005] Betsch, J. M. , & Waller, A. (1994). Chaetotaxic nomenclature of the head, thorax and abdomen in Symphypleona (Insecta, Collembola). Acta Zoologica Fennica, 195, 5–12.

[ece311206-bib-0006] Bonet, F. , & Tellez, C. (1947). Un nuevo genero de Esminturidos (Collembola). Revista de la Sociedad Mexicana de Historia Natural, 8, 193–203.

[ece311206-bib-0007] Börner, C. (1901). Zur Kenntnis der Apterygoten‐Fauna von Bremen und der Nachbardistrikte, Beitrag zu einer Apterygoten‐Fauna Mitteleuropas. Abhandlungen Herausgegeben vom Naturwissenschaftlichen Verein zu Bremen, 17, 1–140.

[ece311206-bib-0008] Bretfeld, G. (1990). Chaetotaxy of four species of the genera Heterosminthurus, Bourletiella, Deuterosminthurus and Proprastiopes (Insecta, Collembola, Symphypleona). Zoologische Jahrbücher Abteilung für Systematik, Ökologie und Geographie der Tiere, 117, 441–489.

[ece311206-bib-0009] Bretfeld, G. (1994). The chaetotaxy of the small abdomen of the Symphypleona (Insecta, Collembola) and its phylogenetic interpretation. Acta Zoologica Fennica, 195, 13–17.

[ece311206-bib-0010] Bretfeld, G. (1999). In W. Dunger (Ed.), Symphypleona. Synopses on Palaearctic Collembola, Vol. 2 (pp. 1–318). Staatliches Museum für Natukunde Görlitz.

[ece311206-bib-0011] Brito, R. A. , Lima, E. C. A. , & Zeppelini, D. (2019). Three new species of Collembola (Arthropoda: Hexapoda) from Brazil. Zootaxa, 4700, 401–430. 10.11646/zootaxa.4700.4.1 32229952

[ece311206-bib-0012] Cassagnau, P. (1974). Chétotaxie et phylogénie chez les Collemboles Poduromorphes. Pedobiologia, 14, 300–312.

[ece311206-bib-0013] Christiansen, K. (1966). The genus Arrhopalites (Collembola: Sminthuridae) in the United States and Canada. International Journal of Speleology, 2, 43–73.

[ece311206-bib-0014] Christiansen, K. , & Bellinger, P. (1998). The Collembola of North America north of the Rio Grande (2nd ed.). Grinnell College.

[ece311206-bib-0015] Cipola, N. G. , Oliveira, J. V. L. C. , Bellini, B. C. , Ferreira, A. S. , Lima, E. C. A. , Brito, R. A. , Stievano, L. C. , Souza, P. G. C. , & Zeppelini, D. (2020). Review of eyeless Pseudosinella Schäffer (Collembola, entomobryidae, and lepidocyrtinae) from Brazilian caves. Insects, 11, 194. 32204486 10.3390/insects11030194 PMC7143100

[ece311206-bib-0016] Culik, M. P. , & Zeppelini Filho, D. (2003). Diversity and distribution of Collembola (Arthropoda: Hexapoda) of Brazil. Biodiversity and Conservation, 12, 1119–1143.

[ece311206-bib-0017] de Lima, E. C. A. , Lopes, B. C. H. , Oliveira‐Neto, M. A. , de Mendonça, M. C. , & Zeppelini, D. (2022). Synthesis of Brazilian Entomobryomorpha (Collembola: Hexapoda) with special emphasis on the equatorial oceanic islands and redescription of the first species of Collembola recorded in Brazil. Diversity, 14, 553.

[ece311206-bib-0018] de Sá Júnior, A. , de Carvalho, L. G. , da Silva, F. F. , & de Carvalho Alves, M. (2012). Application of the Köppen classification for climatic zoning in the state of Minas Gerais, Brazil. Theoretical and Applied Climatology, 108, 1–7.

[ece311206-bib-0019] Deharveng, L. (1983). Morphologie évolutive des Collemboles Neanurinae en particulier de la lignée Neanurienne. Travaux du Laboratoire d'Ecobiologie des Arthropodes Edaphiques, 4, 1–63.

[ece311206-bib-0020] Filser, J. , Faber, J. H. , Tiunov, A. V. , Brussaard, L. , Frouz, J. , De Deyn, G. , Uvarov, A. V. , Berg, M. P. , Lavelle, P. , Loreau, M. , Wall, D. H. , Querner, P. , Eijsackers, H. , & Jiménez, J. J. (2016). Soil fauna: Key to new carbon models. The Soil, 2, 565–582.

[ece311206-bib-0021] Fjellberg, A. (1999). The labial palp in Collembola. Zoologischer Anzeiger, 237, 309–330.

[ece311206-bib-0022] Good, R. (1974). The geography of the flowering plants (4th ed.). Longman Group.

[ece311206-bib-0023] Hopkin, S. P. (1997). Biology of springtails: (Insecta: Collembola). Oxford University Press.

[ece311206-bib-0024] Jordana, R. , & Baquero, E. (2005). A proposal of characters for taxonomic identification of Entomobrya species (Collembola, Entomobryomorpha), with description of a new species. Abhandlungen und Berichte des Naturkundemuseums Görlitz, 76, 117–134.

[ece311206-bib-0025] Jura, C. , Krzysztofowicz, A. , & Kisiel, E. (1987). Embryonic development of *Tetrodontophora bielanensis* (Collembola): Descriptive, with scanning electron micrographs. In H. Andō & C. Jura (Eds.), Recent advances in insect Embryology in Japan and Poland (pp. 77–124). Arthropodan Embryological Society of Japan.

[ece311206-bib-0026] Köppen, W. (1936). Das Geographische System der Klimate. Handbuch der Klimatologie (pp. 7–30). Gebrüder Borntl'aeger.

[ece311206-bib-0027] Lubbock, J. (1862). Notes on the Thysanura. Part I. Smynthuridae. Transactions of the Linnean Society of London, 23, 429–448.

[ece311206-bib-0028] Lubbock, J. (1870). Notes on the Thysanura. Part IV. Transactions of the Linnean Society of London, 27, 277–297.

[ece311206-bib-0029] Lukić, M. , Houssin, C. , & Deharveng, L. (2010). A new relictual and highly troglomorphic species of Tomoceridae (Collembola) from a deep Croatian cave. ZooKeys, 69, 1–16. 10.3897/zookeys.69.739 PMC3088440 21594037

[ece311206-bib-0030] Nayrolles, P. (1988). Chetatoxie Tibiotarsale des Collemboles Symphypleones. Laboratoire d'écobiologie des arthropodes édaphiques de l'Université Paul Sabatier.

[ece311206-bib-0031] Nayrolles, P. (1990a). Chetotaxie Furcale des Collemboles Symphypleones . Entomologiste. ‐ Au Laboratoire d'écobiologie des arthropodes édaphiques de l'Université Paul Sabatier.

[ece311206-bib-0032] Nayrolles, P. (1990b). Chetotaxie de la Base de la Patte des Collemboles Symphypleones . Entomologiste. ‐ Au Laboratoire d'écobiologie des arthropodes édaphiques de l'Université Paul Sabatier.

[ece311206-bib-0033] Nayrolles, P. (1991). La chetotaxie antannaire des Collemboles Symphypleones . Travaux du Laboratoire d'Ecobiologie des Arthropodes Edaphiques.

[ece311206-bib-0034] Packer, L. , Monckton, S. K. , Onuferko, T. M. , & Ferrari, R. R. (2018). Validating taxonomic identifications in entomological research. Insect Conservation and Diversity, 11, 1–12.

[ece311206-bib-0035] Potapov, A. , Bellini, B. , Chown, S. , Deharveng, L. , Janssens, F. , Kováč, Ľ. , Kuznetsova, N. , Ponge, J.‐F. , Potapov, M. , Querner, P. , Russell, D. , Sun, X. , Zhang, F. , & Berg, M. (2020). Towards a global synthesis of Collembola knowledge: Challenges and potential solutions. Soil Organisms, 92, 161–188.

[ece311206-bib-0036] Potapov, A. M. , Guerra, C. A. , van den Hoogen, J. , Babenko, A. , Bellini, B. C. , Berg, M. P. , Chown, S. L. , Deharveng, L. , Kováč, Ľ. , Kuznetsova, N. A. , Ponge, J. F. , Potapov, M. B. , Russell, D. J. , Alexandre, D. , Alatalo, J. M. , Arbea, J. I. , Bandyopadhyaya, I. , Bernava, V. , Bokhorst, S. , … Scheu, S. (2023). Globally invariant metabolism but density‐diversity mismatch in springtails. Nature Communications, 14, 674. 10.1038/s41467-023-36216-6 PMC9905565 36750574

[ece311206-bib-0037] Potapov, M. (2001). Isotomidae. In W. Dunger (Ed.), Synopses on palearctic Collembola (pp. 1–603). Görlitz, German.

[ece311206-bib-0038] Richards, W. R. (1968). Generic classification, evolution, and biogeography of the Sminthuridae of the world (Collembola). Memoirs of the Entomological Society of Canada, 100, 3–54.

[ece311206-bib-0039] Rusek, J. (1998). Biodiversity of Collembola and their functional role in the ecosystem. Biodiversity and Conservation, 7, 1207–1219.

[ece311206-bib-0040] Salmon, J. T. (1964). An index to the Collembola. Royal Society of New Zealand.

[ece311206-bib-0041] Shear, J. A. (1966). A set‐theoretic view of the kÖppen dry climates. Annals of the Association of American Geographers, 56, 508–515.

[ece311206-bib-0042] Szeptycki, A. (1972). Morpho‐systematic studies on Collembola III. Body chaetotaxy in the first instars of several genera of the Entomobryomorpha. Acta Zoologica Cracoviensia, 17, 341–372.

[ece311206-bib-0043] Szeptycki, A. (1979). Chaetotaxy of the Entomobryidae and its phylogenetical significance ‐ Morpho‐systematic studies on Collembola IV. Polska Akademia Nauk.

[ece311206-bib-0044] Tomizuka, S. , & Machida, R. (2015). Embryonic development of a collembolan, Tomocerus cuspidatus Börner, 1909: With special reference to the development and developmental potential of serosa (Hexapoda: Collembola, Tomoceridae). Arthropod Structure & Development, 44, 157–172. 25579205 10.1016/j.asd.2014.12.004

[ece311206-bib-0045] Yosii, R. (1960). Studies on the collembolan genus Hypogastrura. American Midland Naturalist, 64, 257.

[ece311206-bib-0046] Zeppelini, D. , & Brito, R. A. (2014). Two new species of Pararrhopalites (Collembola: Symphypleona: Sminthuridae) in Brazil. Florida Entomologist, 97, 1733–1744.

[ece311206-bib-0047] Zeppelini, D. , Dal Molin, A. , Lamas, C. J. E. , Sarmiento, C. , Rheims, C. A. , Fernandes, D. R. R. , Lima, E. F. B. , Silva, E. N. , Carvalho‐Filho, F. , Kováč, Ľ. , Montoya‐Lerma, J. , Moldovan, O. T. , Souza‐Dias, P. G. B. , Demite, P. R. , Feitosa, R. M. , Boyer, S. L. , Weiner, W. M. , & Rodrigues, W. C. (2021). The dilemma of self‐citation in taxonomy. Nature Ecology & Evolution, 5, 2. 33173203 10.1038/s41559-020-01359-y

[ece311206-bib-0048] Zeppelini, D. , Lima, E. C. A. , Brito, R. A. , & Soares, G. A. (2018). A new species of Pararrhopalites Bonet & Tellez (Collembola, Symphypleona, Sminthuridae) from iron caves in Brazil. Neotropical Entomology, 47, 492–501. 29335870 10.1007/s13744-017-0569-0

[ece311206-bib-0049] Zeppelini, D. , Oliveira, J. V. L. C. , de Lima, E. C. A. , Brito, R. A. , Ferreira, A. S. , Stievano, L. C. , Brito, N. P. , Oliveira‐Neto, M. A. , & Lopes, B. C. H. (2022). Hotspot in ferruginous rock may have serious implications in Brazilian conservation policy. Scientific Reports, 12, 14871. 36050352 10.1038/s41598-022-18798-1 PMC9437091

[ece311206-bib-0050] Zeppelini, D. , Queiroz, G. C. , & Bellini, B. C. (2023). Collembola in Catálogo Taxonômico da Fauna do Brasil . PNUD. Retrieved June 14, 2023, from http://fauna.jbrj.gov.br/fauna/faunadobrasil/379

[ece311206-bib-0051] Zhang, F. , & Deharveng, L. (2015). Systematic revision of Entomobryidae (Collembola) by integrating molecular and new morphological evidence. Zoologica Scripta, 44, 298–311.

